# An approach to identify microRNAs involved in neuropathic pain following a peripheral nerve injury

**DOI:** 10.3389/fnins.2014.00266

**Published:** 2014-08-29

**Authors:** Monica Norcini, Alexandra Sideris, Lourdes A. Martin Hernandez, Jin Zhang, Thomas J. J. Blanck, Esperanza Recio-Pinto

**Affiliations:** ^1^Department of Anesthesiology, NYU Langone Medical CenterNew York, NY, USA; ^2^Department of Neuroscience and Physiology, NYU Langone Medical CenterNew York, NY, USA; ^3^Department of Biochemistry and Molecular Pharmacology, NYU Langone Medical CenterNew York, NY, USA

**Keywords:** peripheral nerve injury, dorsal root ganglia, microRNA, allodynia, neuropathic pain

## Abstract

Peripheral nerve injury alters the expression of hundreds of proteins in dorsal root ganglia (DRG). Targeting some of these proteins has led to successful treatments for acute pain, but not for sustained post-operative neuropathic pain. The latter may require targeting multiple proteins. Since a single microRNA (miR) can affect the expression of multiple proteins, here, we describe an approach to identify chronic neuropathic pain-relevant miRs. We used two variants of the spared nerve injury (SNI): Sural-SNI and Tibial-SNI and found distinct pain phenotypes between the two. Both models induced strong mechanical allodynia, but only Sural-SNI rats maintained strong mechanical and cold allodynia, as previously reported. In contrast, we found that Tibial-SNI rats recovered from mechanical allodynia and never developed cold allodynia. Since both models involve nerve injury, we increased the probability of identifying differentially regulated miRs that correlated with the quality and magnitude of neuropathic pain and decreased the probability of detecting miRs that are solely involved in neuronal regeneration. We found seven such miRs in L3-L5 DRG. The expression of these miRs increased in Tibial-SNI. These miRs displayed a lower level of expression in Sural-SNI, with four having levels lower than those in sham animals. Bioinformatic analysis of how these miRs could affect the expression of some ion channels supports the view that, following a peripheral nerve injury, the increase of the seven miRs may contribute to the recovery from neuropathic pain while the decrease of four of them may contribute to the development of chronic neuropathic pain. The approach used resulted in the identification of a small number of potentially neuropathic pain relevant miRs. Additional studies are required to investigate whether manipulating the expression of the identified miRs in primary sensory neurons can prevent or ameliorate chronic neuropathic pain following peripheral nerve injuries.

## Introduction

Surgical procedures are often associated with chronic neuropathic pain (Macrae, 2001). Despite the introduction of improved surgical procedures, about 10–50% of patients develop chronic pain and 2–10% of patients develop severe disabling chronic pain (Nathan and Pappas, 2003; Kehlet et al., 2006; Pokorny et al., 2008; Vadivelu et al., 2008; Gahm et al., 2010). While the intensity of acute post-operative pain is a good predictor of long-term pain, adequate control of acute post-operative pain does not always prevent the development of chronic pain (Perkins and Kehlet, 2000).

The primary sensory neurons are the first neurons in the sensory pathway to be directly affected following peripheral nerve injury. The injury-evoked changes include alterations in neurotransmitter release, expression of channels and receptors, which contribute to neuronal hyperexcitability and ectopic discharges. These peripheral changes initiate a cascade of events in the central nervous system leading to a decrease in inhibitory control and increase in sensitivity that eventually result in chronic neuropathic pain (Wall and Gutnick, 1974a,b; Wall and Devor, 1983; Tal and Eliav, 1996; Ali et al., 1999; Liu et al., 1999, 2000a,b; Woolf and Mannion, 1999; Wu et al., 2001, 2002; Gold et al., 2003). Many studies have focused on discerning which of the injury-evoked changes in primary sensory neurons contribute to the development of chronic pain. Peripheral nerve injury alters the expression of hundreds of proteins in the dorsal root ganglia (DRG) where the somata of primary sensory neurons are located (Hokfelt et al., 1994; Araki et al., 2001; Bonilla et al., 2002; Costigan et al., 2002; Wang et al., 2002; Xiao et al., 2002; Jimenez et al., 2005; Nilsson et al., 2005; Komori et al., 2007; Stam et al., 2007; Persson et al., 2009a). Some of these changes appear to contribute to the development of neuropathic pain (Persson et al., 2009a; Berger et al., 2011; Xu and Yaksh, 2011). This information has led to successful treatments for acute pain, which usually involve the targeting of several molecules, but sadly effective chronic neuropathic pain treatment still remains elusive. Since nerve injury evokes global increases in gene expression (Araki et al., 2001; Bonilla et al., 2002; Costigan et al., 2002; Wang et al., 2002; Xiao et al., 2002; Jimenez et al., 2005; Nilsson et al., 2005; Komori et al., 2007; Stam et al., 2007), treatment of chronic neuropathic pain may require the simultaneous alteration of multiple proteins. One approach that has been investigated is to produce a general block of protein synthesis; while this approach has shown encouraging results in animal models (Melemedjian et al., 2011) it has limitations in clinical settings because ongoing protein synthesis is required for normal cell function as well as functional recovery following an injury. Another approach is to manipulate the expression of microRNAs (miRs). miRs are small (~22 nucleotides) non-coding RNAs that regulate gene expression by interacting with sites in the 3′ untranslated regions of mRNA molecules to inhibit translation of proteins (Lewis et al., 2005; Chen and Rajewsky, 2007). Since its interaction with mRNA does not require a full complementary sequence, a single miR can target hundreds of distinct mRNA (Lewis et al., 2005). A change in just one miR could theoretically coordinate multiple protein changes that lead to the development of peripheral nerve-injury induced chronic pain. Because of this, there is a recent growing interest in understanding the roles of miRs in pain (Niederberger et al., 2011). The capacity of miRs to affect the translation of multiple pain-relevant proteins make them potential targets for therapeutic intervention to prevent or treat chronic neuropathic pain following peripheral nerve injuries induced by surgery or trauma. Alterations in a large number of miRs expressed both in the DRG and in the nerve stump have been reported in various sciatic nerve injury models (Strickland et al., 2011; von Schack et al., 2011; Yu et al., 2011b; Zhang et al., 2011; Zhou et al., 2011) and in cancer and inflammatory models (Sakai and Suzuki, 2014) of chronic pain. The down-regulation of some of these miRs has been linked to the up-regulation of proteins in the pro-nociceptive machinery (von Schack et al., 2011). Differential expression of mRNAs (Persson et al., 2009a) and miRs (Bali et al., 2014) in DRG have also been postulated to contribute to pain predisposition following spinal nerve injury.

In this study we first describe the simultaneous use of Sural-SNI and Tibial-SNI in adult rats to facilitate the identification of molecular changes that are pain relevant. Using this approach we identified seven miRs that were differentially regulated in DRG. Finally, based on the predicted targets of these miRs, we discuss how their differential expression could be contributing to either the transition from acute to chronic pain in the Sural-SNI model or to the recovery from acute pain in the Tibial-SNI model.

## Materials and methods

### Animal model

Adult male Sprague–Dawley rats (250–300 g) were used following the guidelines approved by the New York University Langone Medical Center Institutional Animal Care and Use Committee. Under isoflurane anesthesia, the spared-nerve injury (SNI) was performed as previously described (Decosterd and Woolf, 2000). We used two different variations of the SNI: the Sural-SNI that consisted in the ligation and cutting of the common peroneal and the tibial nerve branches and the Tibial-SNI that consisted in the ligation and cutting of the common peroneal and the sural nerve branches. The ligated branches were transected distal to the ligature, and 2–3 mm of each distal nerve branch stump was removed. In sham controls, the sciatic nerve and its branches were exposed but none of the branches were ligated or transected.

### Behavioral test

Mechanical allodynia was evaluated in individual rats placed in plexiglass boxes upon an elevated metal grid allowing access to the plantar surface of the hind-paws. Mechanical thresholds were measured in the plantar surface of the hind-paws ipsilateral and contralateral to the injury with an electronic von Frey apparatus equipped with a size 15 filament fitted on the 800 gram arm (IITC Life Sciences, Inc.). Cold allodynia was assessed on the plantar surface of the hind-paws by placing 20 μl of absolute acetone with an eppendorf multistepper pipette and measuring the duration of paw withdrawal in the 30 s immediately following the acetone application. As a control, we also applied 20 μl of water at 37°C before and after the acetone test. Water did not induce a withdrawal response. For each day, mechanical and cold allodynia measurements were repeated three times with an interval of about 5 min between stimuli, and for each animal the mean value was used. Rats were “marked” on the top of their tails and were randomly placed into the individual plexiglass boxes. The investigator doing the measurements could not see the “mark” on the rat's tail. Mechanical thresholds were measured in both the Tibial and Sural areas in all the animals. The person applying the filament was different from the person doing the read out on the electronic von Frey apparatus.

### Lumbar DRG collection

Under isoflurane anesthesia a transcardial perfusion was performed using cold oxygenated artificial cerebro spinal fluid (ACSF) (in nM: Dextran 0.4, Sucrose 125, Glycerol 125, NaHCO_3_ 26, Glucose 15, Hepes 2.1, KCl 3, MgSO_4_ 1.3, KH_2_PO_4_ 1.2; pH 7.4). This was done to cool down the tissues prior to isolation to decrease RNA degradation/processing and to decrease the contribution of blood-derived (plasma and cells) miRs. The rats were then decapitated and their DRG (L3-L5) collected, the DRG's roots removed. Immediately after isolation the DRG were placed in RNAlater stabilization Reagent (Qiagen, cat#1017980) at 4°C for 24 h and subsequently stored at −20°C.

### Total RNA extraction from single DRG

Since the amount of our starting material is very small (1–2 mg) we used a strategy described in the miRNeasy mini Handbook “for purification of total RNA, including miRNA” p. 24 (https://www.dkfz.de/gpcf/fileadmin/downloads/miRNA/Qiagen_miRNeasy_Mini_Handbook.pdf). To inactivate RNAses, the DRG were disrupted using 700 μl of Phenol/guanidine-based QlAzol Lysis reagent (Qiagen, cat#79306) and an automatic cordless motor pestle mixer (cat#K749540-000, Kimble Chanse Kontes). The lysate was then passed through a QlAshredder column (Qiagen, cat#79654). The extraction of total RNA was done by using a phase separation with chloroform. Chloroform (140 μl) was added to the homogenate and the organic and aqueous phases were separated through centrifugation (12,000 g, for 15 min at 4°C). The aqueous upper phase containing RNA was collected and 1.5 × Volume of 100% absolute ethanol was added. It was then passed through an RNeasy Min Elute Spin Colum (that comes within the RNeasy Plus Micro Kit, Qiagen, cat#74034; no other components of the kit were used); and washed one time with 700 μl of RTW buffer and twice with 500 μl of RPE buffer (Total RNA was then eluted from the column with 14–20 μl of RNase free water. The concentration of total RNA was obtained by using a Thermoscientific NanoDrop 2000 Spectrophotometer. The integrity of the samples was evaluated using the Agilent 2100 Bioanalyzer. The Nanodrop and Bioanalyzer measurements were carried out by the NYU Langone Genome Technology Center (NIH/NCI P30CA016087). The RNA integrity number (RIN) of our samples ranged between 7.3 and 9.

### Microarray analysis

We used TaqMan Rodent Array Micro RNA Card-Av 2 (cat#4398967, Applied Biosystems, Life Technologies) in a 7900HT instrument from Applied Biosystems following the company indications (http://tools.lifetechnologies.com/content/sfs/manuals/4399721c.pdf) and performed by the NYU Langone Genome Technology Center. Briefly the probes used were labeled with FAM™ dye linked to their 5′ end. The probes also contained an nonfluorescent quencher (NFQ) at their 3′ end. “When the oligonucleotide probe is intact, the proximity of the quencher dye to the reported dye causes the reported dye signal to be quenched.” Forty cycles of amplification were done, no cut-off value was used. For analysis only miRs that were detected in the 12 samples (12 arrays) were used (Supplement 1). This card contains 384 TaqMan® MicroRNA Assays that enables quantitation of 335 and 226 miRs for mouse and rat, respectively. Many of the mature miRs are identical between mouse and rats. MammU6-4395470, snoRNA135-4380912 and U87-4386735 were used as the reference miRs to obtain ΔC_T_ values for each of the miRs in each of the samples by using ExpressionSuite Software v1.0.3 (Applied Biosystems) and exported to an excel file. In the excel file, the “Sham ΔC_T_” values (the control sample) was used to obtain the “fold change” (2^−ΔΔCT^) of each miR in the experimental groups (Sural-SNI and Tibial-SNI). This was done by subtracting the values between the corresponding DRG (e.g., “L3-DRG-Sural-SNI ΔC_T_” minus “L3-DRG-Sham ΔC_T_,” “L4-DRG-Sural-SNI ΔC_T_” minus “L4-DRG-Sham ΔC_T_” etc.) (Schmittgen and Livak, 2008). There was no outlier exclusion used. To more easily visualize the decreases vs. the increases in expression, the data were presented as “log_2_ (fold change).” The data from Tibial-SNI and Sural-SNI were compared using one-tailed unpaired *t*-test (Prism, GraphPad Software, Inc.), differences with *P* values less than *P* = 0.09 were considered significant and the actual *P* values are given in figure legends. The microarray data were deposited to GEO (http://www.ncbi.nlm.nih.gov/geo/query/acc.cgi?acc=GSE60033).

### Reverse transcription (RT) and quantitative PCR (qPCR) of selected miRs

Stem-loop RT primers and primers to do the corresponding qPCR for the following miRs: miR-1-3p, (ID#002064), miR-130a-3p (ID#000454), miR-133b-3p (ID#002247), miR-143-3p (ID#000466), miR-145-5p (ID#002278), miR-193b-3p (ID#002467), miR-191a-5p (ID#002299), miR-325-3p (ID#002510), miR-335 (ID#000546), U6 snRNA (ID#001973), U87 (ID#001712) and snoRNA135 ID#001230) were purchased from Applied Biosystems (TaqMan microRNA Assays cat#PN4427975). According to the company these primers are the same ones as those used in the TagMan Rodent Array MicroRNA Card Av 2 that we used for the initial microarray analysis. A multiplex RT primer pool was done by mixing 10 μl from each of the 12 individual 5X RT stem-loop primers with 880 μl of 1XTE buffer. The RT step was done using the TaqMan MicroRNA Reverse Transcription Kit (Applied Biosystems, cat# 4366596) in an Eppendorf Mastercycler ep Gradient S with a ramp speed of 2.3°C/s. For each tube the total reaction volume was 15 μl, which included 6 μl of RT stem-loop primer pool, 3 μl of total RNA (350–500 ng for reaction). A water control (3 μl of water) was also run to check the background related with the multiplex of RT stem loop primers. All reactions were run in duplicate The thermal-cycling conditions used were those suggested by the company (30 min at 16°C, 30 min at 42°C, 5 min at 85°C and hold at 4°C at the end).

TargetScan (http://www.targetscan.org/vert_50/) and Miranda (http://www.microrna.org/microrna/getMirnaForm.do) software were used to identify potential mRNA targets for the identified miRs. The list of potential mRNA targets was filtered by selecting those that encode for various ion channels, and are known to be important for normal neuronal excitability.

### Labeling of injured neurons

2–3 crystals (diameter 0.1–0.5 mm) of the tracer DiI (1,1′-dioctadecyl3,3,3′,3′-tetramethylindocarbocyanin-perchlorate) were placed on the stump of the transected branches (30 min) to allow for tracer uptake. Gelfoam was used to support the stumps (of the injured branches), to hold the crystals in place, and to cover the spared (uninjured) nerve branch. Following 30 min, Gelfoam and whatever was left of the crystals were removed. DRG were collected at day 86 post-surgery and sections were obtained as previously described (Castillo et al., 2013). Neuronal somata were visualized by staining the slices with Nissl #N21480 (1:100; Invitrogen Molecular Probes) (20 min, RT) according to the manufacturer's protocol. Dil and Nissl labeling was visualized by using Images captured with a ZeissAxiovert 200 (Germany) inverted microscope equipped with fluorescence and Nomarski optics, objective 10×.

### Inter-digital distance

Animals were held such that the hind limbs were left undisturbed and allowed to move freely. The distance between digit 1 and 5 was measured. For each group of animals, the values were normalized to the value observed in the Sham-CL paw.

### Statistical analysis

To compare more than two groups we used One-Way ANOVA, followed by a Bonferroni's Multiple comparison Post-test. To compare only two groups, we used Unpaired *t*-test, one-tailed. These tests were done using the Prism 5.0.4 for Windows (GraphPad Software, Inc.).

## Results

### Different patterns of mechanical- and cold-allodynia in sural- and tibial SNI

**Figure 1:**
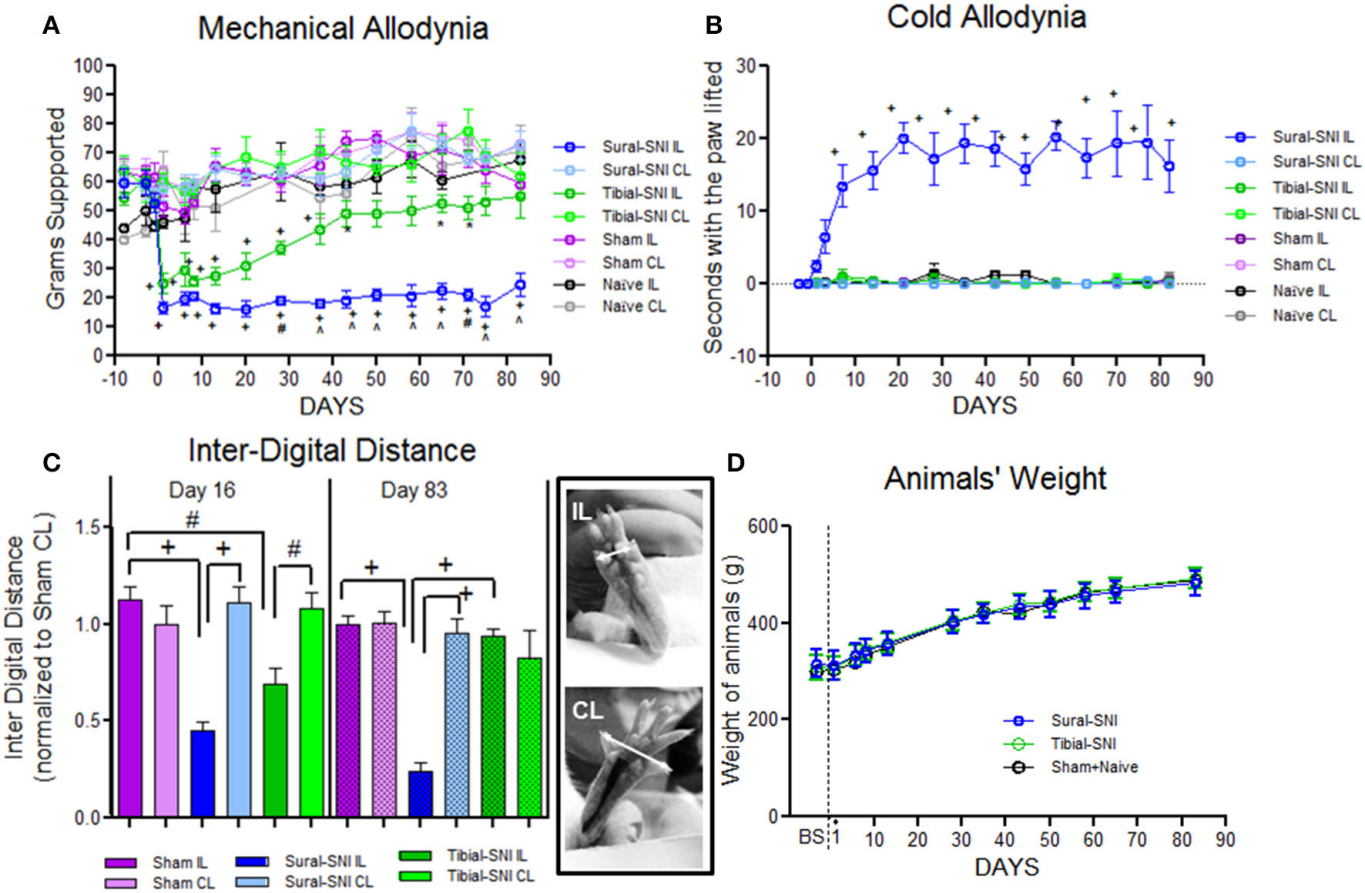
**Tibial-SNI and Sural-SNI display differences in their pain phenotype. (A)** Mechanical allodynia measurements in the ipsilateral (IL) and contralateral (CL) paws of Sural-SNI, Tibial-SNI, Sham and Naïve rats. Only the Sural-SNI and Tibial-SNI groups showed responses that were significant different between their IL and CL paws: ^+^*P* < 0.001, ^*^P < 0.05. In addition, a significant difference was found in the IL responses between Sural-SNI and Tibial-SNI starting at Day 28 post-surgery: ^∧^*P* < 0.001, ^#^*P* < 0.01. For Sural-SNI and Tibial-SNI, the number of rats was *n* = 10 for Days -8, -2, and -1 pre-surgery, and for Days 1, 6, 8, 13, and 28 post-surgery; *n* = 7 for Days 20, 37, 43, 50, and 58 post-surgery; *n* = 6 for Day 65 post-surgery, *n* = 4 for days 71, 75 post-surgery, and *n* = 5 for day 83 post-surgery. For Sham, *n* = 8 from Day -8 pre-surgery to Day 28 post-surgery, and *n* = 5 for the rest of the post-surgery days. For Naïve, *n* = 3 for all time points. **(B)** Cold allodynia measurements in the IL and CL paws of Sural-SNI, Tibial-SNI, Sham and Naïve rats. The only group that showed significance difference between the IL and CL responses was the Sural-SNI ^+^*P* < 0.001. The number of rats was, *n* = 4 for Sural-SNI and Sham, and *n* = 3 for Tibial-SNI and Naïve. **(C)** Inter-digital distance measurements in the IL and CL paws. Values were normalized to the mean value observed in the Sham-CL paw. Significant difference between the indicated groups: ^+^*P* < 0.001, ^#^*P* < 0.01. The number of rats for Day 16 post-surgery were: *n* = 9 for Sham and Sural-SNI, and *n* = 8 for Tibial-SNI; for Day 83 post-surgery: *n* = 6 for all groups. For **(A–C)**: One-Way ANOVA *P* < 0.0001; Bonferroni's Multiple comparison Post-test. **(D)** Animal's weight gain was comparable between Sham+Naive, Sural-SNI, and Tibial-SNI animals. The number of rats was *n* = 6 for all groups.

We selected two modalities of the spared nerve injury (SNI) of the sciatic nerve, the Sural-SNI and the Tibial-SNI. In the two SNI variants, the onset of mechanical allodynia and its highest allodynia value (lowest threshold value) was detected on Day 1 post-surgery and it was of a similar magnitude in both SNI variants (Figure 1A). However, behavioral discrepancies emerged over time. The Sural-SNI animals maintained strong mechanical allodynia throughout the entire observation period of 85 days (Figure 1A, dark blue line), consistent with previous reports (Decosterd and Woolf, 2000; Suter et al., 2003); however, the Tibial-SNI animals started to recover from mechanical allodynia at about 2 weeks post-surgery, and displayed almost full recovery by day 40 post-surgery (Figure 1A, dark green line) (Recio-Pinto et al., 2012). The naïve and sham operated animals, showed no significant decrease in the threshold for mechanical allodynia (Figure 1A, black and purple lines, respectively). Measurements in the contralateral paws were comparable for all the groups (Figure 1A, corresponding light color lines).

We found that the Sural-SNI animals developed cold allodynia (evidenced by an increase in duration of paw withdrawal) as previously reported (Decosterd and Woolf, 2000), while the Tibial-SNI animals never developed cold allodynia. In Sural-SNI animals the onset of mechanical allodynia was faster than the onset of cold allodynia, and mechanical allodynia reached its peak at an earlier time than cold allodynia (Day 1 vs. Day 20 post-surgery) (Figures 1A,B).

**Figure 2:**
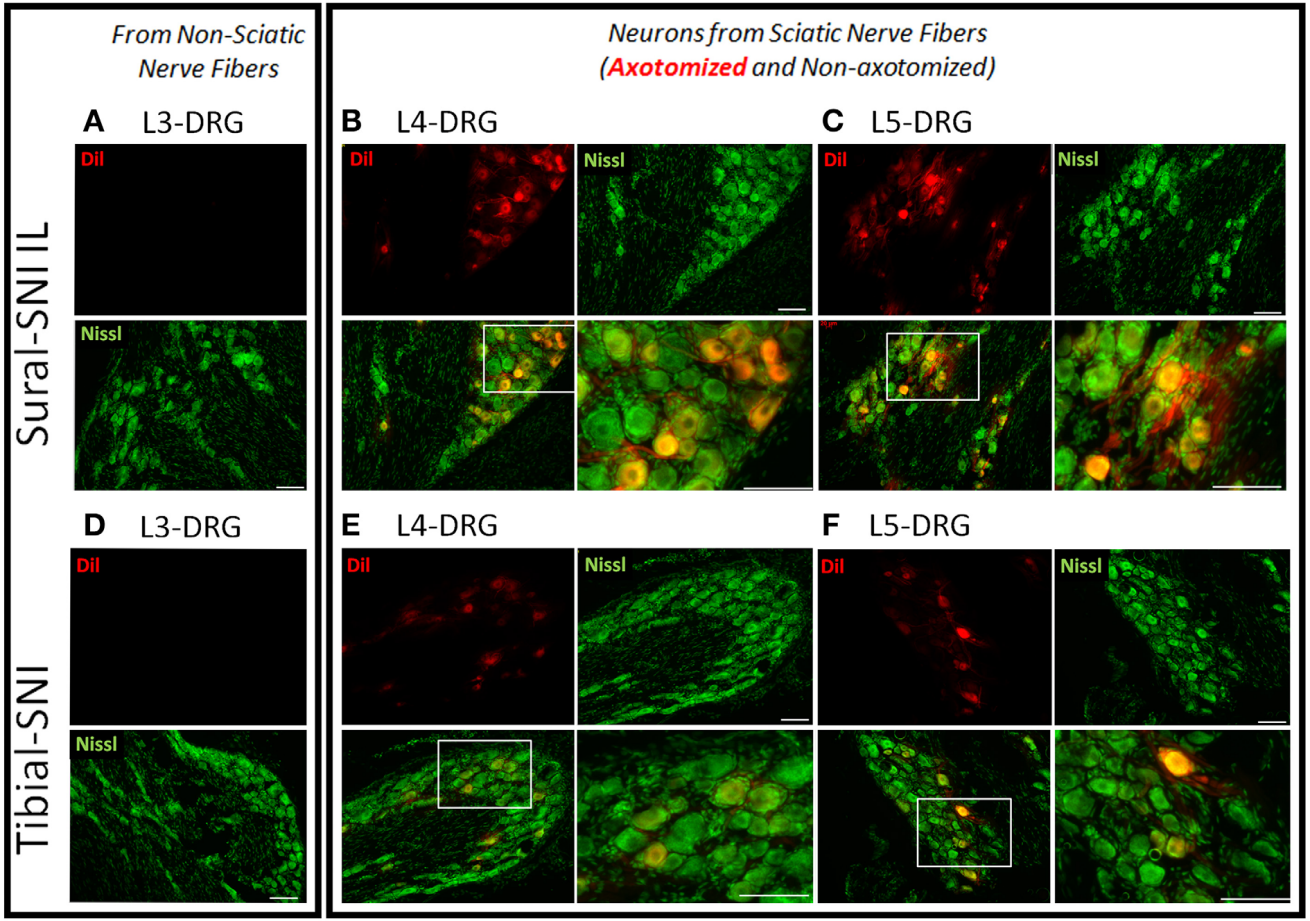
**Labeling of injured neurons**. Images of DRG sections from ipsilateral L3-, L4-, and L5-DRG derived from Sural-SNI **(A–C)** and Tibial-SNI **(D–F)**. Sensory neurons are labeled with Nissl (green) and injured sensory neurons are labeled with Dil (red). Sensory neurons of various sizes are damaged in L4- and L5-DRG, but not in L3-DRG. A larger proportion of injured primary sensory neurons are observed in Sural-SNI than in Tibial SNI. White boxes in the bottom left panels of **(B,C,E,F)** correspond to the expanded area shown in their corresponding bottom right panels. Scale bars = 100 μm.

The inter-digital distance in the ipsilateral paw significantly decreased following both SNI modalities, and the magnitude of change was not statistically different between Sural-SNI and Tibial-SNI at Day 16 post-surgery (Figure 1C). By Day 83 post-surgery, the decrease in inter-digital distance was maintained in the Sural-SNI, but it was resolved in Tibial-SNI (Figure 1C). Animals in all the groups showed comparable increases in weight over time (Figure 1D) indicating that the surgery did not limit the animals' access to and interest in food. In both SNI modalities primary sensory neurons of various sizes are damaged in L4- and L5-DRG, but not in L3-DRG; however a larger proportion is damaged in Sural-SNI than in Tibial SNI (Figure 2).

### Changes in microRNA expression in DRG following SNI

Since the Sural-SNI and Tibial-SNI models involve damage to the sciatic nerve, but only the former develops chronic pain, we used this behavioral difference to identify miRs for which their dysregulation within the DRG correlated with the development of chronic pain following a peripheral nerve injury. The sensory innervation of the rat hind paw involves fibers from the sciatic and saphenous nerves. The allodynia that develops from cutting some of the sciatic nerve branches is evoked by stimulation of the nerve terminals from the spared sciatic nerve branches as well as from the saphenous nerve that becomes hyperexcitable as a result of interactions with the injured fibers (Kingery and Vallin, 1989; Kingery et al., 1993; Ro and Jacobs, 1993; Attal et al., 1994; Tal and Bennett, 1994; Guilbaud et al., 1995; Sotgiu and Biella, 1997; Smith et al., 2013). In rat, the sensory neurons of the saphenous nerve are located in the L3-DRG and for the sciatic nerve mostly in the L4- and L5-DRG. Hence, we isolated total RNA from individual L3-DRG, L4-DRG and L5-DRG (ipsilateral and contralateral) to be used for miR analysis.

To identify the miRs that were differentially dysregulated between Tibial-SNI and Sural-SNI, we first performed 12 microarrays in a limited number of samples (in four individual DRG per group: Sham, Tibial-SNI and Sural-SNI; two L3-DRG and two L4-DRG). Then, miRs identified as having differential expression were corroborated with real time qRT-PCR in RNA isolated from individual DRG (L3, L4, and L5) derived from 4 rats per group (groups: Sham, Sural-SNI and TIbial-SNI).

**Figure 3:**
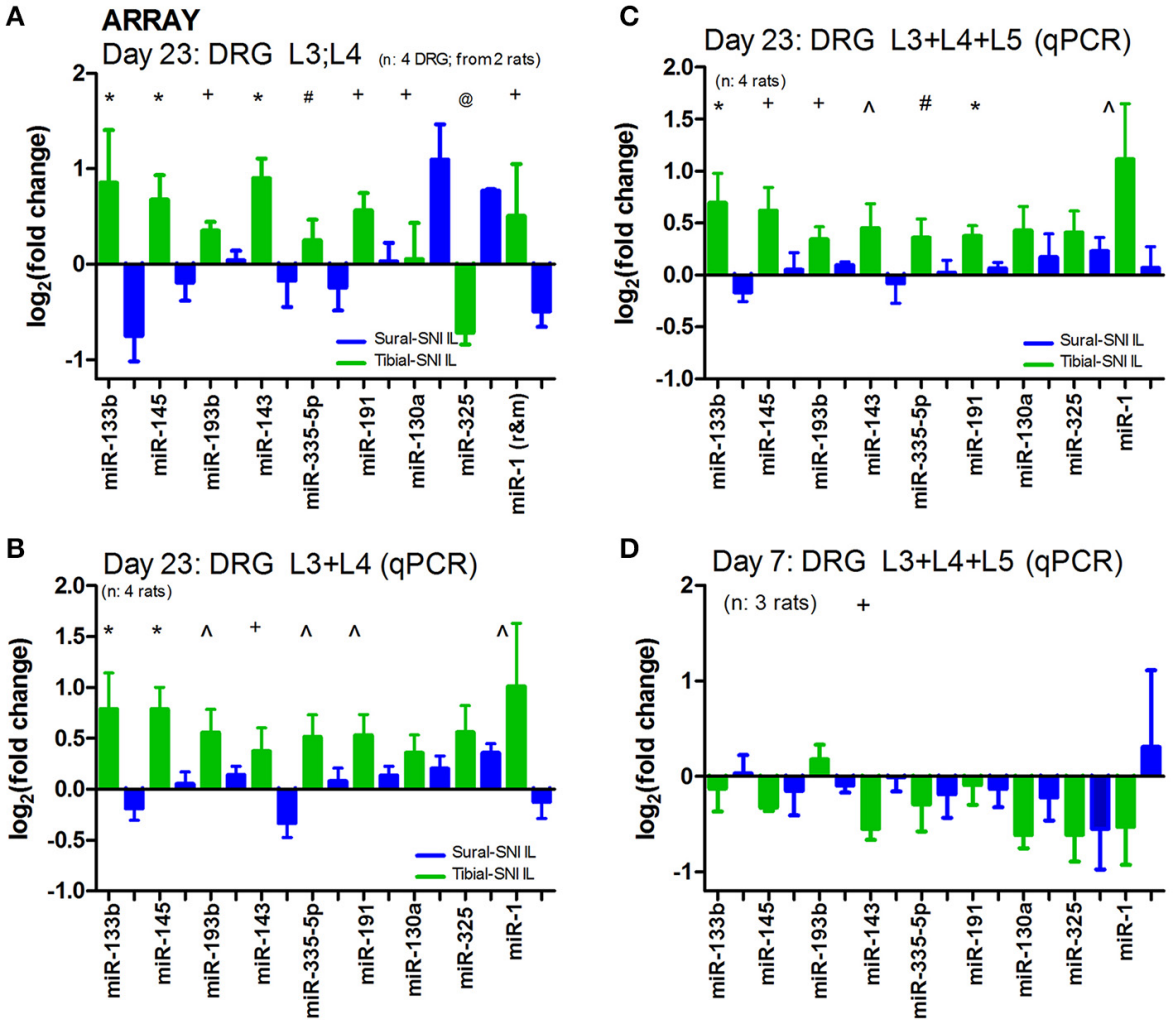
**In ipsilateral DRG seven miRs are differentially regulated between Sural-SNI and Tibial-SNI. (A)** By using a microarray chip containing probes for detecting 375 miRs, we found that only nine miRs were differentially regulated in IL DRG between Sural-SNI and Tibial-SNI at Day 23. Ct values were <30.60 for miR-133b, <27.78 for miR-145, <27.66 for miR-193b, <30.19 for miR-143, <33.69 for miR-335, <23.42 for miR-191, <37.89 for miR-130a, <36.00 for miR-325, <32.04 for miR-1. Reference miRs were: MammU6-4395470 (Ct values < 19.30), snoRNA135-4380912 (Ct values < 33.98), and U87-4386735 (Ct values < 28.32). The *n* value was four DRG (two L3 and two L4). For each SNI-group, the mean value from four individual DRG (two L3-DRG and two L4 DRG) were used. **(B,C)** By using qPCR it was confirmed that seven out of the initially identified nine miRs (in **A**) were differentially regulated in IL DRG between Sural-SNI and Tibial SNI at Day 23, whether using the data from L3 and L4 **(B)** or the data from L3, L4, and L5 **(C)**; *n* = 4 rats. **(D)** By using qPCR the levels of the identified miRs were measured at Day 7; *n* = 3 rats. **(B–D)** The Ct values were <29.94 for miR-133b, <24.67 for miR-145, <27.42 for miR-193b, <34.17 for miR-143, <30.74 for miR-335, < 23.90 for miR-191, <32.17 for miR-130a, <32.62 for miR-325, <30.61 for miR-1. Reference miRs were: snoRNA135-4380912 (Ct values < 30.94) and U87-4386735 (Ct values < 25.16). For each rat, the mean value between their DRG (L3, L4, and L5) was used. In **(A–D)**, the “fold change” (2^−ΔΔCT^) was obtained by comparing the experimental sample (Sural-SNI or Tibial-SNI ΔC_T_) vs. the sham sample (Sham ΔC_T_). Significance between the Tibial-SNI and Sural-SNI: ^@^*P* < 0.005, ^*^P < 0.02, ^+^*P* < 0.05, ^∧^*P* < 0.07; ^#^*P* < 0.09, Unpaired *t*-test, one-tailed. Hence the Sham value will be set to “0” (log2(fold change)=0) (in this and in Figures 4, 5).

DRG were isolated at Day 23 following the injury since this time point was associated with 50% recovery in mechanical allodynia in Tibial-SNI (Figure 1A). We postulated that at Day 23 changes were occurring which allow for recovery in Tibial-SNI. Some of these changes may be transitory, such that they may be turned off upon reaching maximal recovery (in Tibial-SNI). Hence, by selecting Day 23, we are increasing the probability of detecting not only the changes that may contribute to the development of chronic neuropathic pain in Sural-SNI, but also changes that may contribute to the recovery from post-nerve injury pain in Tibial-SNI. Moreover, by using DRG we are concentrating on measuring the changes that occur in the sensory neurons. The microarray used had probes for 380 endogenous miRs; of these, about 190 were detected in DRG. Comparison between the two injury models reveals that only nine miRs were differentially dysregulated between Sural and TIbial-SNI (Figure 3A). The simultaneous analysis of Sural-SNI vs. Tibial-SNI refines the miRs that are potentially relevant to the differences in the pain phenotype of both SNI variants. Of the nine differentially regulated miRs, in Tibial-SNI seven showed an increase and one a decrease, while in Sural-SNI five showed a decrease and two an increase. When using qPCR we confirmed that seven out of the nine miRs identified with the microarrays displayed significant differential regulation between Tibial- and Sural-SNI whether when using data from L3 and L4 (Figure 3B) or when using data from L3, L4 and L5 (Figure 3C). In both the microarrays (Figure 3A) and qPCR (Figures 3B,C) seven miRs (miR-133b, miR-145, miR-193b, miR-143, miR-335-5p, miR-191, miR-1) had a significantly higher level of expression in Tibial-SNI than in Sural-SNI. The results for the other two miRs (miR-130a and miR-325) observed in the microarray were not confirmed with qPCR. This was the case even when using the same RNA samples. We used two different batches of primers for qPCR and the results were the same. We do not understand this discrepancy, however, since they did not show a significant difference between Tibial-SNI and Sural-SNI when using qPCR we did not consider them in our subsequent analysis. At Day 7, the expression of eight of the nine miRs showed no statistical difference between Sural-SNI and Tibial-SNI (Figure 3D), at a time when both SNI modalities display the same level of mechanical allodynia. At Day 7, the only miR that displayed a difference (between Sural-SNI and Tibial-SNI) was miR-143, which showed a downregulation in Tibial-SNI. At Day 23 miR-143 shows an upregulation in Tibial-SNI. The sequence of five of the seven identified miRs is identical between mice, rats and humans. The two exceptions, miR-1 and miR-193b are identical between mice and humans. In rats miR-1 differs in one nucleotide, and miR-193b is 3 nucleotides shorter (Supplement 2).

**Figure 4:**
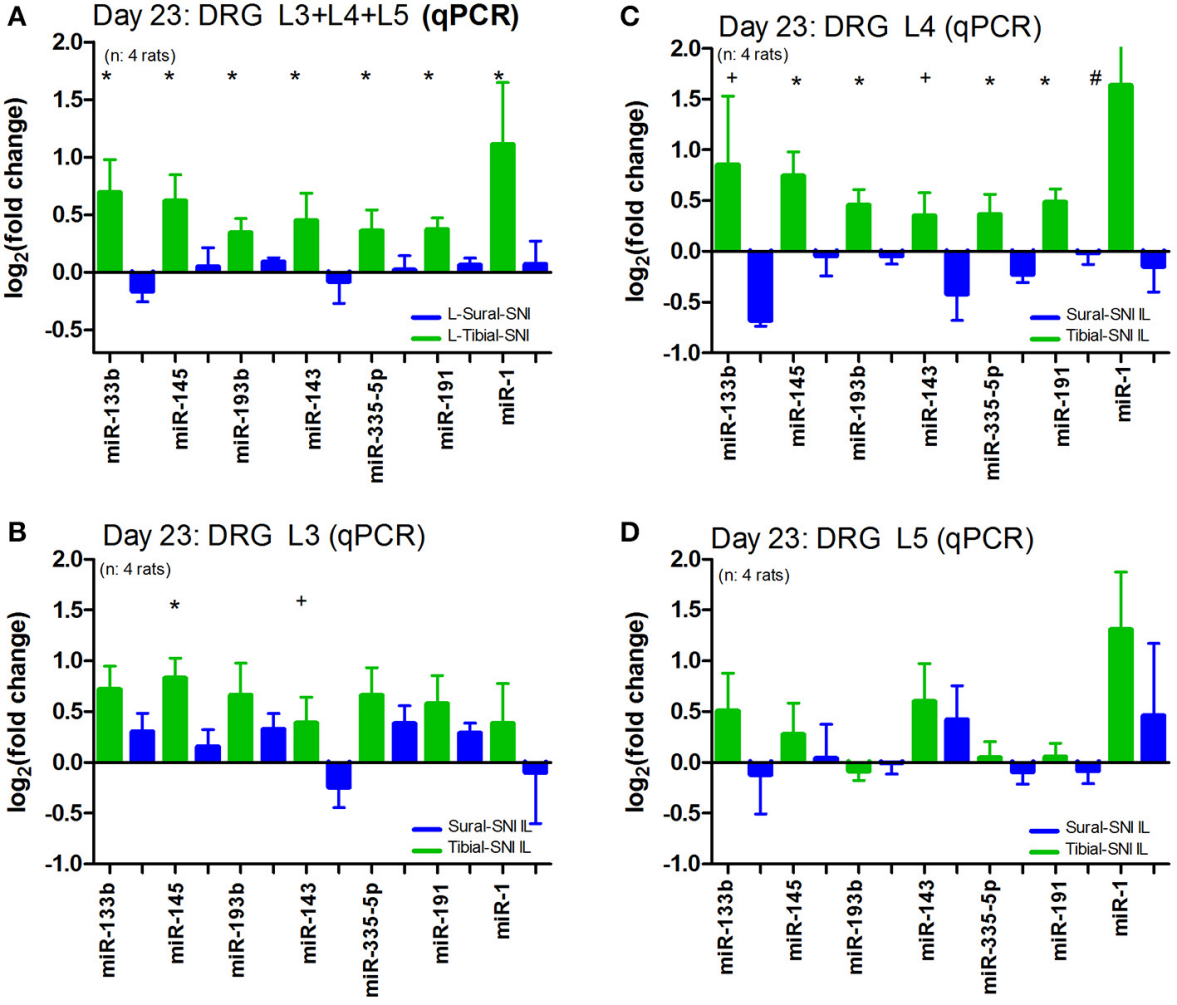
**The level of miR differential regulation is highest in ipsilateral L4-DRG as compared to ipsilateral L3-DRG and L5-DRG**. For comparison purposes the expression level of the seven identified miRs is shown when the data of the three IL DRG (L3, L4, and L5) was pooled together **(A)**; and for the individual IL DRG: **(B)** L3-DRG; **(C)** L4-DRG; and **(D)** L5-DRG. The data in **(A)** corresponds to that shown in Figure 3B, except that only the seven miRs with statistical significant difference between Sural-SNI IL vs. Tibial-SNI IL are shown In all cases *n* = 4 rats. ^*^P < 0.02, ^+^*P* < 0.05, ^#^*P* < 0.09 between Sural-SNI IL vs. Tibial-SNI IL, Unpaired *t*-test, one-tailed.

We further analyzed the data by plotting the levels of expression of these miRs in each DRG (Figure 4). Of the three DRG examined, L4-DRG had the largest magnitude of differential regulation of these miRs between Tibial-SNI and Sural-SNI (Figure 4B). In L4-DRG not only was there an increase in the expression of these seven miRs in Tibial-SNI, but there was also a decrease in four of these miRs in Sural-SNI (Figure 4B). L3-DRG, which contains the somata of the uninjured saphenous nerve fibers, also showed differential regulation between the two SNI variants, but only three of these miRs (miR-133b, miR-145, miR-143) were significantly different. In L5-DRG, although a similar tendency was observed (higher expression levels in Tibial-SNI than in Sural-SNI), none of the seven miRs, showed a significant differential regulation between Tibial-SNI and Sural-SNI.

**Figure 5:**
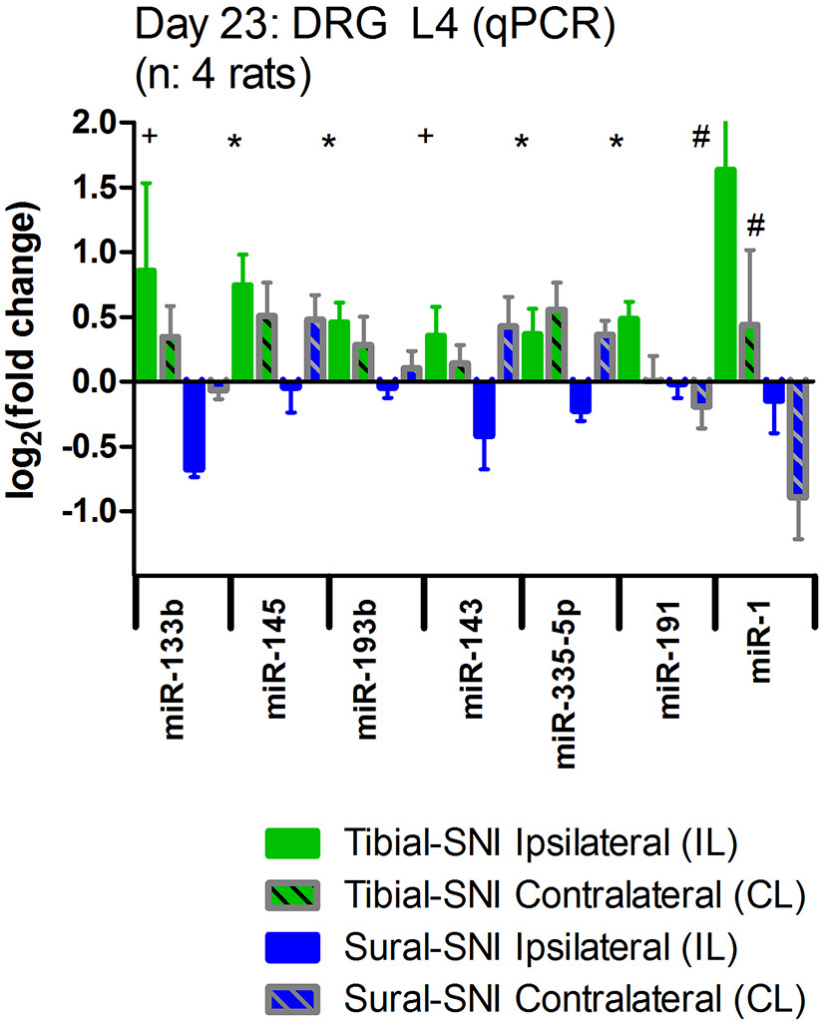
**Nerve injury also evokes changes in miR expression in the contralateral DRG**. The expression level of the seven identified miRs is shown for the ipsilateral L4-DRG (IL) and from the contralateral L4-DRG (CL) derived from Sural-SNI and Tibial-SNI. The seven miRs showed differential regulation in the ipsilateral L4-DRG between the two SNI variants (as shown in Figure 4C). Compared to values of DRG derived from Sham animals (which in the graph corresponds to 0) these seven miRs also showed changes in regulation in the contralateral L4-DRG. These changes were comparable in the two SNI variants, only being significantly different for miR-1. Significant difference between Sural-SNI L vs. Tibial SNI-L or between Sural-SNI CL vs. Tibial-SNI CL: ^*^P < 0.02, ^+^*P* < 0.05, ^#^*P* < 0.09, Unpaired *t*-test, one-tailed.

We also examined the expression of these seven miRs in the contralateral DRG. We found that as compared to sham, the expression of these miRs was also affected in the contralateral DRG (Figure 5). However, except for miR-1, the regulation in the contralateral DRG was comparable in the two SNI variants. A similar pattern was observed for contralateral L5-DRG and contralateral L3-DRG (data not shown).

**Figure 6:**
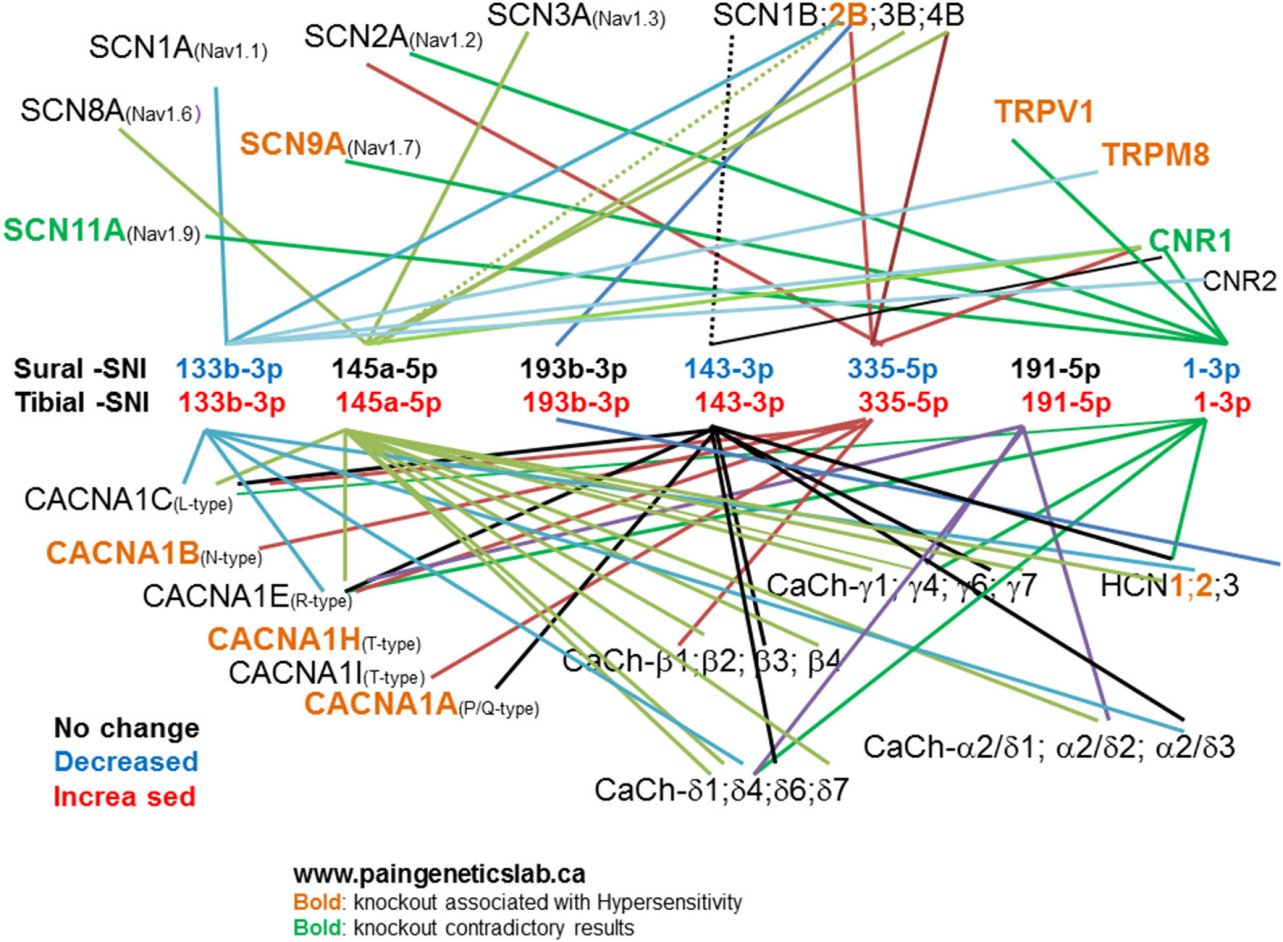
**Predicted mRNA targets for the identified miRs**. TargetScan and Miranda software was used to identify potential mRNA targets for the identified miRs. The list of potential mRNA targets was filtered: by selecting those that encode for ion channels. Here we show those encoding for voltage-dependent sodium channels: α (SCN**X**A) and β (SCN**X**B) subunits; voltage-dependent calcium channels: α (CACNA1**X**), β (CaCh-β **X**), γ (CaCh- γ**X**), γ2δX (CaCh-γ2δX), and δ (CaCh-δX) subunits. The pacemaker channels (HCN**X**), the ligand-gated, non-selective cation channel TRPV1, the transient receptor potential channel TRPM8, and cannabinoid receptors (CNR**X**). “**X**” Stands for various numbers as indicated in the figure. We then used the http://www.jbldesign.com/jmogil/enter.html (Lacroix-Fralish et al., 2007) to find which mRNA targets have been associated with neuronal hypersensitivity by using knockout studies.

To investigate the pain relevance of the seven identified miRs we checked their predicted mRNA targets using TargetScan and Miranda software. Since alterations in neuronal excitability of both injured (Wall and Gutnick, 1974a,b; Wall and Devor, 1983; Tal and Eliav, 1996; Liu et al., 1999, 2000a,b) and uninjured neurons (Ali et al., 1999; Wu et al., 2001, 2002; Gold et al., 2003; Smith et al., 2013) are believed to contribute to the initiation and maintenance of chronic neuropathic pain following peripheral nerve injury, we selected mRNA targets that encode for ion channels known to contribute and modulate neuronal excitability and some that have been already postulated to contribute to chronic neuropathic pain. This was done to investigate how the observed changes in miR expression could potentially affect neuronal excitability following injury. Figure 6 shows some of these mRNA targets, the miR name is color-coded to indicate whether its expression did not change (black), decreased (blue), or increased (red), in DRG derived from either the Sural-SNI (top row) or Tibial-SNI (bottom row). Pooled data and that for L4-DRG (Figures 4A,C) were used. Based on knockout studies, the predicted mRNA targets (shown in light brown) have already been associated with hypersensitivity in various pain models (http://www.jbldesign.com/jmogil/enter.html) (Lacroix-Fralish et al., 2007), including some in which alterations in mechanical and/or cold allodynia were found. These include the voltage dependent sodium channel SCN9A (Nassar et al., 2004), the sodium channel beta 2 subunit (SCN2B) (Pertin et al., 2005; Lopez-Santiago et al., 2006), the voltage-dependent calcium channels T-type (Choi et al., 2007; Lee et al., 2009; Chen et al., 2010), N-type (Hatakeyama et al., 2001; Kim et al., 2001; Saegusa et al., 2001) and P/Q-type (Luvisetto et al., 2006), the pacemaker channels HCN1 (Momin et al., 2008) and HCN2 (Emery et al., 2011), the ligand-gated, non-selective cation channel TRPV1 (Caterina et al., 2000; Wang et al., 2008) and the transient receptor potential channel TRPM8 (Bautista et al., 2007; Dhaka et al., 2007; Gentry et al., 2010; Knowlton et al., 2010). Some of these targets such as the voltage-dependent calcium channels T-type (Latham et al., 2009; Lee et al., 2014) and N-type (Shao et al., 2012; Adams and Berecki, 2013), and the TRPM8 channel (Knowlton et al., 2011) were originally identified using pharmacological studies. In studies measuring protein expression levels and using pharmacological agents proteins encoded by other of the identified mRNA targets, such as the voltage-dependent sodium channels SCN8A (Deuis et al., 2013), the auxiliary α2δ-1 subunit (Moore et al., 2009, 2011; Bauer et al., 2010) and β (3) subunit (Li et al., 2012) of voltage-gated calcium channels, have also been implicated in the development of either mechanical or cold allodynia.

Based on our miR results, the translation of these mRNAs would be predicted to increase in the Sural-SNI due to the decreased expression of four of the identified miRs and would be predicted to decrease in the Tibial-SNI due to the increased expression of all of the seven identified miRs.

## Discussion

In this study we describe an experimental approach to increase the probability of detecting biological changes that contribute to the development of chronic neuropathic pain following a peripheral nerve injury. The approach consisted in using the Sural-SNI and Tibial-SNI, because both models involved sciatic nerve damage but displayed distinct pain phenotypes. Both SNI modalities displayed the same level of mechanical allodynia and functional disruption as ascertained by the reduction of inter-digital distance in the acute and sub-chronic post-operative period. However, over time, only the Tibial-SNI recovered in both of these phenotypes. Moreover, only the Sural-SNI developed chronic cold allodynia. By using this approach we identified seven miRs within the DRG that are likely transducers of these two distinct pain phenotypes, in part, via regulation of neuronal excitability and neuronal sprouting.

Peripheral nerve injury alters the expression of hundreds of proteins in DRG (Hokfelt et al., 1994; Araki et al., 2001; Bonilla et al., 2002; Costigan et al., 2002; Wang et al., 2002; Xiao et al., 2002; Jimenez et al., 2005; Nilsson et al., 2005; Komori et al., 2007; Stam et al., 2007). The differences in protein expression between models (Niederberger et al., 2008), in part, reflect that not all of the injury-induced protein changes necessarily lead to neuropathic pain. The Sural-SNI model is one of two models that involve partial sciatic nerve transection in which blocking the nerve impulses before and during a week following the injury does not eliminate the development of chronic neuropathic pain (Dougherty et al., 1992; Suter et al., 2003, 2011; Yang et al., 2014). This phenomenon resembles the outcome of a significant number of surgical patients for whom control of acute post-surgical pain does not prevent the development of chronic neuropathic pain (Perkins and Kehlet, 2000). At least in this specific patient population, the development of chronic pain involves dysregulation of multiple proteins that have not yet been fully identified.

Because a single miR can affect the expression of many proteins (Klein et al., 2005) we used the two SNI variants to increase the probability of identifying pain relevant miRs in the DRG. We found that only a few miRs were differentially regulated in DRG derived from Tibial-SNI and Sural-SNI. The relatively low number of miRs that were differentially regulated was expected, since by using the two SNI modalities the probability of including injury-induced changes in miR expression that did not contribute to the development of chronic pain was most likely reduced.

The allodynia that develops following the SNI injury (ligation and cut of two sciatic nerve branches with removal of 2–3 mm of nerve stump) is evoked by stimulation of the spared nerve terminals that become hyperexcitable as a result of interactions with the injured fibers, as has been shown for the spared-sural branch in Sural-SNI (Smith et al., 2013). Moreover, functional interactions between the saphenous nerve and the damaged sciatic nerve contribute to pain disorders following sciatic nerve injury (Kingery and Vallin, 1989; Kingery et al., 1993; Ro and Jacobs, 1993; Attal et al., 1994; Tal and Bennett, 1994; Guilbaud et al., 1995; Sotgiu and Biella, 1997). Evoked (mechanical, cold) injury-induced neuropathic pain reflects changes in primary sensory neurons, even if other components such as motor and sympathetic are playing a role. Differences in injury-evoked inflammation could also be contributing to our results, because inflammation not only can lead to neuropathic pain, but also to changes in miR expression in DRG (Sakai and Suzuki, 2014). Based on our findings, it appears that depending on the proportion and/or type of damaged primary sensory neurons, the damaged neurons and those closely interacting with them undergo changes in miR expression that may either limit the development of chronic neuropathic pain as in Tibial-SNI or promote the development of chronic neuropathic pain as in Sural-SNI.

Since injury-induced neuronal hyperexcitability contributes to the initiation and maintenance of chronic neuropathic pain following a peripheral nerve injury (Wall and Gutnick, 1974a,b; Wall and Devor, 1983; Tal and Eliav, 1996; Ali et al., 1999; Liu et al., 1999, 2000a,b; Wu et al., 2001, 2002; Gold et al., 2003), we inspected how the observed changes in miR expression could potentially affect the expression of the predicted mRNA targets that encode for ion channels. This analysis indicated that the translation of various ion channels, including some that have already been found to contribute to neuronal hyperexcitability and neuropathic pain (see Results), would be expected to increase in Sural-SNI primary sensory neurons due to the decreased expression of at least four miRs (miR-133b-3p, miR143, miR-335-5p, miR-1). On the other hand, the translation of these ion channels would be predicted to decrease in the Tibial-SNI primary sensory neurons due to the increase in expression of all seven identified miRs (miR-133b, miR-145, miR-193b, miR-143, miR-335-5p, miR-191, miR-1). At Day 7 the two SNI modalities display the same high level of mechanical allodynia. Interestingly, at Day 7 the expression of these miRs in both SNI modalities is similar and would be expected to increase excitability since most of miRs show an expression level lower than that displayed by Sham controls. This analysis supports the view that the down-regulation of four of the identified miRs appears to orchestrate the development of chronic neuropathic pain in Sural-SNI while the up-regulation of all seven of the identified miRs appear to orchestrate the recovery from post-nerve injury induced pain in Tibial-SNI.

We found that as compared to sham, the expression of these miRs was also affected in the contralateral DRG (Figure 5). Except for miR-1, the regulation in the contralateral DRG was comparable in the two SNI variants, and more closely resembles the expression pattern in the ipsilateral DRG of Tibial-SNI. This expression pattern in the contralateral DRG correlates with the observed lack of allodynia and with the predicted lack of an increase in excitability in the contralateral paw. Hence, because miR changes are occurring in the contralateral DRG, which may reflect a compensatory or protective effect, using the contralateral DRG as the control should be avoided, even when using peripheral nerve injury models in which neuropathic pain does not develop in the contralateral paw.

Of the seven miRs identified, miR-145 is consistently altered in two other sciatic nerve injury models. Following complete sciatic nerve transection, miR-145 is decreased in DRG (Zhang et al., 2011; Zhou et al., 2011) and nerve stump (Yu et al., 2012). Sciatic nerve transection results in chronic mechanical hyperalgesia and autotomy (Kingery and Vallin, 1989; Persson et al., 2009b). Following sciatic nerve crush, miR-145 is decreased in the nerve stump (Viader et al., 2011) at a time period in which animals display mechanical and cold allodynia (Tamaddonfard et al., 2014). In our dual injury model, the Tibial-SNI animals, but not the Sural-SNI animals, recovered from post-injury pain and showed an increase in miR-145. Taken together, an increase in miR-145 correlates with recovery from post-nerve injury induced pain, while a lack of an increase (Sural-SNI) or decrease (sciatic nerve transection, sciatic nerve crush) of miR-145 correlates with development of chronic pain following a peripheral nerve injury. Enhancement of miR-145 reduces neurite outgrowth in cultured DRG sensory neurons (Zhang et al., 2011). Hence, it appears that decreasing miR-145 in primary sensory neurons contributes to the development of chronic pain by enhancing the expression of ion channels known to contribute to neuronal hyperexcitability and possibly by promoting non-specific sprouting following a peripheral nerve injury. The opposite will be the case in Tibial-SNI in which there is an increase in miR-145.

Another miR that has been studied in reference to neuropathic pain is miR-1. In DRG, the level of miR-1 was increased following axotomy while it was decreased following partial sciatic nerve ligation (Kusuda et al., 2011). Enhancement of miR-1 reduces neurite outgrowth of cultured DRG sensory neurons (Bastian et al., 2011). Based on our findings we propose that the observed decrease of miR-1 in partial nerve ligation (Kusuda et al., 2011) and Sural-SNI (this study) might contribute to the development of chronic pain by enhancing excitability and by promoting aberrant sprouting of nerve branches. In contrast, the increase in miR-1 following axotomy (Kusuda et al., 2011) and Tibial-SNI (this study) could actually be a compensatory response to limit the level of neuronal hyperexcitability and the level of aberrant sprouting, at least during the first weeks following axotomy. These may contribute to recovery in Tibial-SNI. Consistent with this view the overexpression of miR-1 in cardiac myocytes slows conduction and depolarizes these cells partly through post-transcriptional repression of GJA1 (which encodes for connexin-43) (Yang et al., 2007). Moreover, an increase in connexin-43 expression in trigeminal DRG appears to contribute to pain development following nerve injury (Ohara et al., 2008). In Sural-SNI, the protein level of Nav1.7 channel has been found to be increased (Laedermann et al., 2013) despite a decrease in Nav1.7 mRNA (Berta et al., 2008; Laedermann et al., 2014). These apparent contradictory results could be explained by our observation that miR-1 is decreased. The decrease in miR-1 would accelerate protein translation compensating for the decrease in mRNA.

The pacemaker channels HCN1 and HCN2 contribute to the generation of ectopic firing (Jiang et al., 2008) and neuropathic pain (Momin et al., 2008; Wan, 2008; Emery et al., 2011) following sciatic nerve injury. We found that several of the identified miRs that were decreased in Sural-SNI and increased in Tibial-SNI are predicted to target mRNA encoding for these channels (Figure 5). Decrease of these miRs could account for the previously observed increase in pacemaker channels and hyperexcitability of primary sensory neurons following sciatic nerve injury.

Peripheral nerve injury changes neuronal activity not only on primary sensory neurons but also in ascending and descending spinothalamic and spinomesencephalic tract neurons (Martin and Ewan, 2008; Lavertu et al., 2014; Salter, 2014). Moreover, whether initiated by a peripheral or a central injury, chronic neuropathic pain ultimately becomes centralized through alteration (synapses, morphology, receptors, channels, excitability, and function) of various brain systems; the extent of these changes varies between chronic pain conditions. For example, the resulting altered sensation, such as allodynia and hyperalgesia, involve changes in the primary sensory and motor cortices, the thalamus and posterior Insula (Moisset and Bouhassira, 2007). Hence targeting primary sensory neurons may be mostly helpful at stages or in conditions in which their altered function is contributing to the initiation or to the continuous maintenance of the central alterations that result in chronic neuropathic pain.

Peripheral nerve injury not only produces changes in miR expression at the DRG, but also at the spinal cord (Genda et al., 2013) and brain (Arai et al., 2013; Hori et al., 2013). Hence, pain relevant miRs at any sensory level would potentially provide additional tools for the treatment of chronic neuropathic pain. With respect to the Sural-SNI model, an increase in miR-124a at the spinal cord leads to a decrease in mechanical allodynia (Willemen et al., 2012). Moreover, depending on the nerve injury type the relevant miRs may differ. For example, in the spinal nerve ligation model, intrathetal injections of miR-21 (Sakai and Suzuki, 2013), miR-103 (Favereaux et al., 2011), miR-183 (Lin et al., 2014) or of miR-195 inhibitor (Shi et al., 2013), and injection of miR-7a into the L5-DRG (Sakai et al., 2013) attenuate pain. In a bone cancer model, intrathetal injections of miR-1-inhibitor or miR-34c-5p-inhibitor, respectively produce a mild or a strong decrease in mechanical hypersentitivity (Bali et al., 2013). In spinal cord injury, inthathecal injections of miR-23b attenuate mechanical and thermal pain (Im et al., 2012). In inflammatory pain models, intravenous delivery (Kynast et al., 2013) or intrathecal delivery (Willemen et al., 2012) of miR-124a ameliorates pain.

It has been reported that the expression of 19–200 miRs is altered in DRG following peripheral nerve injuries (Strickland et al., 2011; Yu et al., 2011a,b; Zhang et al., 2011; Zhou et al., 2011; Li et al., 2013); such variability has been observed even when using the same injury model (Yu et al., 2011a,b; Zhang et al., 2011; Zhou et al., 2011). However, what is most surprising is the lack of overlap in the identified miRs among studies (Strickland et al., 2011; Yu et al., 2011a,b; Zhang et al., 2011; Zhou et al., 2011; Li et al., 2013). Since DRG isolation is time and labor intensive we perfused the animals with cold ACSF to reduce RNA degradation and processing during tissue isolation and to remove blood-derived miR contaminants. As far as we can tell, none of the previously published studies referred to above used perfusion prior to DRG isolation. Therefore, the lack of overlap in altered miRs, particularly when using the same injury model may be a consequence of methodological issues ranging from blood miR contaminants and metabolic shock-evoked RNA processing, to tissue isolation time.

Six of the seven miRs that we identified were within the list of 48 miRs altered in the sciatic nerve stump following a sciatic nerve crush (Viader et al., 2011). Isolation of the nerve stump is much faster, and hence, pre-cooling prior to tissue isolation may not be as critical for RNA stability/processing as when isolating DRG. Local protein synthesis also occurs in axons (Murashov et al., 2007) and neuromas (mass of nerve tissue resulting from abnormal regrowth of the stumps of severed nerves) (Huang et al., 2008) and miRs at these locations (Natera-Naranjo et al., 2010; Kaplan et al., 2013) are likely to also regulate local protein synthesis. Therefore, it is not surprising that six of the miRs that we identified to be differentially regulated in DRG were also found to be altered in the sciatic nerve following a sciatic nerve crush (Viader et al., 2011). By using the rationale from our dual SNI variants we interpret that the decrease in expression of four miRs (miR-133b-3p, miR-145, miR-143, and miR-1) contributes to pain while the increase in expression of two miRs (miR-193b-3p and miR-191-5p) limits the level of pain that develops following a sciatic nerve crush. In the array, additional potential differentially dysregulated miRs were detected, that similarly displayed an apparent higher level of expression in Tibial-SNI than in Sural-SNI (Supplement 1). However, based on their predicted targets the ones we further corroborated with qPCR where those targeting multiple ion channels (Figure 6).

There are several limitations in the present study, one being that the predicted mRNA targets were not confirmed and this will require both mRNA measurements and functional studies. Obviously the selected mRNA (Figure 5) would not be the only pain-relevant mRNA targets. In fact, we expect that the effect of the identified miRs on many more mRNA targets would be required to underlie the processes leading to the different pain phenotypes. Our goal was to identify likely pain-relevant miRs by using the two SNI models, and to validate their pain relevance by analyzing how their expression changes would affect neuronal excitability. By comparing the two SNI modalities, rather than Sham vs. injury, we are increasing the probability of detecting miR changes that involve aspects of neuronal processes (excitability, protein expression, regeneration) that contribute to their different pain phenotype. Measurements are needed to determine which of the observed changes in miR expression at Day 23 are transitory and which ones are sustained. Additional studies are required to investigate whether manipulating the expression of the identified miRs in primary sensory neurons can prevent or ameliorate sustained neuropathic pain following peripheral nerves injury.

In this study we first described behavioral differences in the two SNI variants, and how these can potentially be used to identify peripheral-nerve injury induced changes (molecular, functional, or structural) at any level of the pain-sensory pathways (DRG, spinal cord, brain) that correlate with the development of neuropathic pain. Second, we described the simultaneous use of these two models to identify pain relevant miRs in the DRG; and by doing so we identified seven miRs that are likely transducers of these two pain phenotypes. Finally, to validate our approach, the analysis focused on interpreting how changes in the identified miRs could contribute to neuronal hyperexcitability. We also discussed how previously published results can be interpreted in light of our findings using the dual model approach. In summary, the data analysis supports the view that the down-regulation of four miRs (miR-133b, miR-143, miR-335-5p, miR-1) appears to orchestrate the development of chronic neuropathic pain in Sural-SNI while the up-regulation of seven miRs (miR-133b, miR-145, miR-193b, miR-143, miR-335-5p, miR-191, miR-1) appear to orchestrate the recovery from post-nerve injury induced pain in Tibial-SNI.

### Conflict of interest statement

The authors declare that the research was conducted in the absence of any commercial or financial relationships that could be construed as a potential conflict of interest.
