# Supplementary material for: An approach to identify microRNAs involved in neuropathic pain following a peripheral nerve injury
Source: Front Neurosci. 2014 Aug 29;8:266. doi: 10.3389/fnins.2014.00266 (PMC4148822; doi:10.3389/fnins.2014.00266)
Supplement: Supplementary file 1 [file DataSheet1.PDF]

The statitistics were done with Prism.

S= Sural SNI; T=Tibial-SNI

Data for miRs that where detected in all the 12 samples (12 arrays).
Values given as: log2(fold change)
“Sham ∆CT” values (the control sample) was used to obtain the “fold change” (2-∆∆CT) of each miR in the experimental groups (Sural-SNI and Tibial-SNI). (See methods for details)

	S-mmu-miR-133b-4395358	T-mmu-miR-133b-4395358	S-mmu-miR-145-4395389	T-mmu-miR-145-4395389
Number of values	4	4	4	4
Minimum	-1.255	-0.223	-0.498	-0.047
25% Percentile	-1.198	-0.05275	-0.4713	0.1365
Median	-0.8605	0.631	-0.3125	0.8545
75% Percentile	-0.191	1.987	0.2108	1.041
Maximum	-0.023	2.381	0.359	1.047
**Mean**	**-0.7498**	**0.855**	**-0.191**	**0.6773**
Std. Deviation	0.5363	1.103	0.3824	0.51
Std. Error	0.2681	0.5516	0.1912	0.255
Lower 90% CI of mean	-1.381	-0.4429	-0.6408	0.07727
Upper 90% CI of mean	-0.1188	2.153	0.2588	1.277
**To compare with SHAM:**				
One sample t test				
Theoretical mean (for Sham)	0	0	0	0
Actual mean	-0.7498	0.855	-0.191	0.6773
Discrepancy	0.7498	-0.855	0.191	-0.6773
90% CI of discrepancy	-1.381 to -0.1188	-0.4429 to 2.153	-0.6408 to 0.2588	0.07727 to 1.277
t, df	t=2.796 df=3	t=1.550 df=3	t=0.9991 df=3	t=2.656 df=3
P value (two tailed)	0.0681	0.2189	0.3914	0.0766
Sum	-2.999	3.42	-0.764	2.709
**Comparing S vs T (Sural-SNI vs Tibial-SNI)**				
Unpaired t test				
P value	0.0199		0.0172	

S-mmu-miR-193b-4395597	T-mmu-miR-193b-4395597	S-mmu-miR-143-4395360	T-mmu-miR-143-4395360	S-mmu-miR-335-5p-4373045	T-mmu-miR-335-5p-4373045
4	4	4	4	4	4
-0.127	0.179	-0.753	0.312	-0.876	-0.333
-0.1115	0.2013	-0.6282	0.4732	-0.7178	-0.1783
-0.0225	0.3105	-0.253	1.063	-0.1915	0.307
0.2547	0.545	0.369	1.174	0.1758	0.6183
0.333	0.609	0.576	1.176	0.281	0.715
**0.04025**	**0.3523**	**-0.1708**	**0.9035**	**-0.2445**	**0.249**
0.2043	0.1853	0.5508	0.4072	0.4781	0.4334
0.1021	0.09266	0.2754	0.2036	0.2391	0.2167
-0.2001	0.1342	-0.8188	0.4244	-0.807	-0.2609
0.2806	0.5703	0.4773	1.383	0.318	0.7589
0	0	0	0	0	0
0.04025	0.3523	-0.1708	0.9035	-0.2445	0.249
-0.04025	-0.3523	0.1708	-0.9035	0.2445	-0.249
-0.2001 to 0.2806	0.1342 to 0.5703	-0.8188 to 0.4773	0.4244 to 1.383	-0.8070 to 0.3180	-0.2609 to 0.7589
t=0.3941 df=3	t=3.801 df=3	t=0.6200 df=3	t=4.437 df=3	t=1.023 df=3	t=1.149 df=3
0.7198	0.032	0.5792	0.0213	0.3817	0.3338
0.161	1.409	-0.683	3.614	-0.978	0.996
0.0322		0.0101		0.0885	

S-mmu-miR-191-4395410	T-mmu-miR-191-4395410	S-mmu-miR-130a-4373145	T-mmu-miR-130a-4373145	S-mmu-miR-325-4395640	T-mmu-miR-325-4395640
4	4	4	4	4	4
-0.449	0.053	0.202	-0.912	0.736	-1.049
-0.366	0.196	0.4118	-0.7297	0.7415	-0.9798
0.0815	0.6645	1.088	0.233	0.769	-0.649
0.3745	0.8353	1.787	0.6572	0.798	-0.5185
0.406	0.879	2.005	0.66	0.804	-0.516
**0.03**	**0.5653**	**1.096**	**0.0535**	**0.7695**	**-0.7158**
0.3894	0.3576	0.7371	0.7551	0.02918	0.2517
0.1947	0.1788	0.3686	0.3776	0.01459	0.1259
-0.4281	0.1445	0.2283	-0.8349	0.7352	-1.012
0.4881	0.986	1.963	0.9419	0.8038	-0.4196
0	0	0	0	0	0
0.03	0.5653	1.096	0.0535	0.7695	-0.7158
-0.03	-0.5653	-1.096	-0.0535	-0.7695	0.7158
-0.4281 to 0.4881	0.1445 to 0.9860	0.2283 to 1.963	-0.8349 to 0.9419	0.7352 to 0.8038	-1.012 to -0.4196
t=0.1541 df=3	t=3.161 df=3	t=2.972 df=3	t=0.1417 df=3	t=52.74 df=3	t=5.686 df=3
0.8873	0.0508	0.059	0.8963	< 0.0001	0.0108
0.12	2.261	4.382	0.214	3.078	-2.863
0.0447		0.0478		< 0.0001	

S-miR-1	T-miR-1	S-rno-miR-1-4395765	T-rno-miR-1-4395765	S-mmu-miR-1-4395333	T-mmu-miR-1-4395333
8	8	4	4	4	4
-1.342	-0.646	-0.842	-0.461	-1.342	-0.646
-0.836	-0.5802	-0.836	-0.4293	-1.052	-0.6395
-0.3005	0.004	-0.5895	0.004	-0.1325	-0.065
-0.1088	1.149	-0.2702	3.017	-0.08425	1.149
-0.084	3.908	-0.24	3.908	-0.084	1.368
**-0.494**	**0.5059**	**-0.5652**	**0.8638**	**-0.4228**	**0.148**
0.4569	1.539	0.3098	2.06	0.6145	0.9705
0.1615	0.5441	0.1549	1.03	0.3072	0.4853
-0.8001	-0.5252	-0.9298	-1.56	-1.146	-0.9938
-0.1879	1.537	-0.2007	3.287	0.3002	1.29
0	0	0	0	0	0
-0.494	0.5059	-0.5652	0.8638	-0.4228	0.148
0.494	-0.5059	0.5652	-0.8638	0.4228	-0.148
-0.8001 to -0.1879	-0.5252 to 1.537	-0.9298 to -0.2007	-1.560 to 3.287	-1.146 to 0.3002	-0.9938 to 1.290
t=3.058 df=7	t=0.9297 df=7	t=3.649 df=3	t=0.8386 df=3	t=1.376 df=3	t=0.3050 df=3
0.0184	0.3835	0.0355	0.4632	0.2626	0.7803
-3.952	4.047	-2.261	3.455	-1.691	0.592
0.05		0.1096		0.1794	

S-rno-miR-532-5p-4395752	T-rno-miR-532-5p-4395752	S-rno-miR-450a-4381124	T-rno-miR-450a-4381124	S-rno-miR-351-4395764	T-rno-miR-351-4395764
4	4	4	4	4	4
-1.374	0.013	-0.331	-0.924	-0.191	0.093
-1.059	0.06275	-0.1815	-0.669	-0.1053	0.1308
0.1355	0.2545	0.437	0.446	0.221	0.442
1.372	1.327	0.9572	0.8163	1.027	0.715
1.701	1.67	1.074	0.823	1.272	0.74
**0.1495**	**0.548**	**0.4042**	**0.1978**	**0.3807**	**0.4292**
1.272	0.7574	0.5914	0.8201	0.6276	0.31
0.6358	0.3787	0.2957	0.41	0.3138	0.155
-1.347	-0.3431	-0.2915	-0.7671	-0.3577	0.0645
1.646	1.439	1.1	1.163	1.119	0.794
0	0	0	0	0	0
0.1495	0.548	0.4042	0.1978	0.3807	0.4292
-0.1495	-0.548	-0.4042	-0.1978	-0.3807	-0.4292
-1.347 to 1.646	-0.3431 to 1.439	-0.2915 to 1.100	-0.7671 to 1.163	-0.3577 to 1.119	0.06450 to 0.7940
t=0.2351 df=3	t=1.447 df=3	t=1.367 df=3	t=0.4823 df=3	t=1.213 df=3	t=2.769 df=3
0.8292	0.2437	0.265	0.6626	0.3118	0.0696
0.598	2.192	1.617	0.791	1.523	1.717
0.3048		0.3485		0.4472	

S-rno-miR-339-3p-4395760	T-rno-miR-339-3p-4395760	S-rno-miR-207-4381096	T-rno-miR-207-4381096	S-rno-miR-196c-4395750	T-rno-miR-196c-4395750
4	4	4	4	4	4
-0.564	-0.615	-2.323	-0.493	0.006	0.049
-0.547	-0.5317	-1.993	-0.3915	0.0395	0.1767
-0.3455	0.0665	-0.585	0.195	0.1725	0.7305
0.5798	0.496	0.308	1.26	0.271	1.429
0.838	0.523	0.467	1.521	0.293	1.605
**-0.1043**	**0.01025**	**-0.7565**	**0.3545**	**0.161**	**0.7787**
0.6483	0.5487	1.205	0.8735	0.1209	0.6527
0.3242	0.2743	0.6025	0.4367	0.06043	0.3263
-0.867	-0.6352	-2.174	-0.6732	0.0188	0.01086
0.6585	0.6557	0.6611	1.382	0.3032	1.547
0	0	0	0	0	0
-0.1043	0.01025	-0.7565	0.3545	0.161	0.7787
0.1043	-0.01025	0.7565	-0.3545	-0.161	-0.7787
-0.8670 to 0.6585	-0.6352 to 0.6557	-2.174 to 0.6611	-0.6732 to 1.382	0.01880 to 0.3032	0.01086 to 1.547
t=0.3216 df=3	t=0.03736 df=3	t=1.256 df=3	t=0.8117 df=3	t=2.664 df=3	t=2.386 df=3
0.7689	0.9725	0.2981	0.4764	0.0761	0.0971
-0.417	0.041	-3.026	1.418	0.644	3.115
0.3982		0.093		0.056	

S-mmu-miR-99b-4373007	T-mmu-miR-99b-4373007	S-mmu-miR-98-4373009	T-mmu-miR-98-4373009	S-mmu-miR-93-4373302	T-mmu-miR-93-4373302
4	4	4	4	4	4
-0.082	-0.747	-0.865	-1.48	-1.536	-1.142
-0.022	-0.5273	-0.7105	-1.232	-1.197	-0.936
0.49	0.189	0.3225	0.4305	0.3065	0.3335
0.8228	0.4785	1.794	2.223	2.553	2.679
0.823	0.556	2.094	2.515	3.14	3.244
**0.4303**	**0.04675**	**0.4685**	**0.474**	**0.5543**	**0.6923**
0.4634	0.5587	1.305	1.795	1.971	1.913
0.2317	0.2793	0.6527	0.8977	0.9854	0.9567
-0.1149	-0.6105	-1.067	-1.638	-1.764	-1.559
0.9754	0.704	2.004	2.586	2.873	2.943
0	0	0	0	0	0
0.4303	0.04675	0.4685	0.474	0.5543	0.6923
-0.4303	-0.04675	-0.4685	-0.474	-0.5543	-0.6923
-0.1149 to 0.9754	-0.6105 to 0.7040	-1.067 to 2.004	-1.638 to 2.586	-1.764 to 2.873	-1.559 to 2.943
t=1.857 df=3	t=0.1674 df=3	t=0.7178 df=3	t=0.5280 df=3	t=0.5625 df=3	t=0.7236 df=3
0.1603	0.8777	0.5247	0.6341	0.6131	0.5216
1.721	0.187	1.874	1.896	2.217	2.769
0.1657		0.4981		0.4616	

S-mmu-miR-92a-4373013	T-mmu-miR-92a-4373013	S-mmu-miR-9-4373285	T-mmu-miR-9-4373285	S-mmu-miR-872-4395375	T-mmu-miR-872-4395375
4	4	4	4	4	4
0.088	0.277	-0.531	-1.181	-0.085	-0.32
0.1398	0.2863	-0.446	-0.9483	-0.0195	-0.2883
0.323	0.4665	0.2675	0.2105	0.293	0.3695
0.9435	0.7938	0.7455	0.9193	0.598	1.294
1.141	0.852	0.752	1.002	0.661	1.414
**0.4688**	**0.5155**	**0.189**	**0.0605**	**0.2905**	**0.4582**
0.4622	0.2717	0.6502	0.9827	0.319	0.85
0.2311	0.1358	0.3251	0.4913	0.1595	0.425
-0.07505	0.1959	-0.5759	-1.096	-0.08475	-0.5418
1.013	0.8351	0.9539	1.217	0.6657	1.458
0	0	0	0	0	0
0.4688	0.5155	0.189	0.0605	0.2905	0.4582
-0.4688	-0.5155	-0.189	-0.0605	-0.2905	-0.4582
-0.07505 to 1.013	0.1959 to 0.8351	-0.5759 to 0.9539	-1.096 to 1.217	-0.08475 to 0.6657	-0.5418 to 1.458
t=2.028 df=3	t=3.795 df=3	t=0.5814 df=3	t=0.1231 df=3	t=1.822 df=3	t=1.078 df=3
0.1356	0.0321	0.6018	0.9098	0.1661	0.3599
1.875	2.062	0.756	0.242	1.162	1.833
0.4336		0.4173		0.3622	

S-mmu-miR-744-4395435	T-mmu-miR-744-4395435	S-mmu-miR-708-4395452	T-mmu-miR-708-4395452	S-mmu-miR-687-4386750	T-mmu-miR-687-4386750
4	4	4	4	4	4
-0.629	-0.287	-0.656	-0.596	-1.276	-1.711
-0.4758	-0.2518	-0.4243	-0.5827	-1.197	-1.534
0.174	0.038	0.346	0.206	-0.746	-0.778
0.439	1.031	1.133	1.222	0.2365	-0.2447
0.464	1.3	1.37	1.311	0.493	-0.142
**0.04575**	**0.2722**	**0.3515**	**0.2818**	**-0.5688**	**-0.8523**
0.4951	0.718	0.8294	0.9939	0.7705	0.6719
0.2475	0.359	0.4147	0.4969	0.3853	0.3359
-0.5367	-0.5725	-0.6243	-0.8875	-1.475	-1.643
0.6282	1.117	1.327	1.451	0.3378	-0.06181
0	0	0	0	0	0
0.04575	0.2722	0.3515	0.2818	-0.5688	-0.8523
-0.04575	-0.2722	-0.3515	-0.2818	0.5688	0.8523
-0.5367 to 0.6282	-0.5725 to 1.117	-0.6243 to 1.327	-0.8875 to 1.451	-1.475 to 0.3378	-1.643 to -0.06181
t=0.1848 df=3	t=0.7584 df=3	t=0.8476 df=3	t=0.5670 df=3	t=1.476 df=3	t=2.537 df=3
0.8652	0.5034	0.4589	0.6104	0.2364	0.0849
0.183	1.089	1.406	1.127	-2.275	-3.409
0.311		0.4588		0.2996	

S-mmu-miR-685-4386748	T-mmu-miR-685-4386748	S-mmu-miR-680-4381079	T-mmu-miR-680-4381079	S-mmu-miR-672-4395438	T-mmu-miR-672-4395438
4	4	4	4	4	4
-1.5	-6.664	-0.8	-1.116	-1.001	-4.653
-1.367	-5.009	-0.4418	-0.745	-0.857	-3.558
-0.848	0.1335	0.663	0.5295	-0.2975	-0.101
-0.1555	1.318	2.846	2.823	0.04	0.5295
0.036	1.654	3.564	3.534	0.11	0.682
**-0.79**	**-1.186**	**1.023**	**0.8692**	**-0.3715**	**-1.043**
0.638	3.725	1.829	1.943	0.4731	2.439
0.319	1.862	0.9147	0.9715	0.2366	1.219
-1.541	-5.568	-1.13	-1.417	-0.9281	-3.912
-0.03945	3.196	3.175	3.155	0.1851	1.826
0	0	0	0	0	0
-0.79	-1.186	1.023	0.8692	-0.3715	-1.043
0.79	1.186	-1.023	-0.8692	0.3715	1.043
-1.541 to -0.03945	-5.568 to 3.196	-1.130 to 3.175	-1.417 to 3.155	-0.9281 to 0.1851	-3.912 to 1.826
t=2.477 df=3	t=0.6367 df=3	t=1.118 df=3	t=0.8948 df=3	t=1.570 df=3	t=0.8556 df=3
0.0895	0.5696	0.3451	0.4368	0.2144	0.4551
-3.16	-4.743	4.09	3.477	-1.486	-4.173
0.4205		0.4562		0.3041	

S-mmu-miR-667-4386769	T-mmu-miR-667-4386769	S-mmu-miR-652-4395463	T-mmu-miR-652-4395463	S-mmu-miR-574-3p-4395460	T-mmu-miR-574-3p-4395460
4	4	4	4	4	4
-3.825	-0.798	-0.184	-0.577	-0.676	-0.239
-3.092	-0.5318	-0.053	-0.328	-0.5488	-0.048
0.026	0.351	0.3505	0.674	-0.0545	0.7235
2.378	0.7088	0.814	1.363	0.2028	0.9895
2.855	0.8	0.965	1.507	0.251	1.012
**-0.2295**	**0.176**	**0.3705**	**0.5695**	**-0.1335**	**0.555**
2.844	0.6864	0.4697	0.8842	0.4	0.5701
1.422	0.3432	0.2349	0.4421	0.2	0.285
-3.575	-0.6315	-0.1821	-0.4707	-0.6041	-0.1157
3.116	0.9835	0.9231	1.61	0.3371	1.226
0	0	0	0	0	0
-0.2295	0.176	0.3705	0.5695	-0.1335	0.555
0.2295	-0.176	-0.3705	-0.5695	0.1335	-0.555
-3.575 to 3.116	-0.6315 to 0.9835	-0.1821 to 0.9231	-0.4707 to 1.610	-0.6041 to 0.3371	-0.1157 to 1.226
t=0.1614 df=3	t=0.5128 df=3	t=1.578 df=3	t=1.288 df=3	t=0.6675 df=3	t=1.947 df=3
0.882	0.6434	0.2128	0.288	0.5522	0.1467
-0.918	0.704	1.482	2.278	-0.534	2.22
0.3955		0.3524		0.0477	

S-mmu-miR-544-4395680	T-mmu-miR-544-4395680	S-mmu-miR-543-4395487	T-mmu-miR-543-4395487	S-mmu-miR-542-5p-4395693	T-mmu-miR-542-5p-4395693
4	4	4	4	4	4
-1.143	-0.791	-2.158	-1.148	-0.613	-2.106
-0.8075	-0.7897	-1.795	-0.9173	-0.5962	-1.556
0.3815	0.088	-0.682	0.331	-0.479	0.29
0.7425	1.185	-0.02825	1.028	0.7093	1.003
0.802	1.259	0.182	1.075	1.083	1.175
**0.1055**	**0.161**	**-0.835**	**0.1472**	**-0.122**	**-0.08775**
0.8685	1.103	0.9717	1.037	0.8077	1.418
0.4342	0.5515	0.4858	0.5183	0.4038	0.7089
-0.9163	-1.137	-1.978	-1.072	-1.072	-1.756
1.127	1.459	0.3082	1.367	0.8282	1.58
0	0	0	0	0	0
0.1055	0.161	-0.835	0.1472	-0.122	-0.08775
-0.1055	-0.161	0.835	-0.1472	0.122	0.08775
-0.9163 to 1.127	-1.137 to 1.459	-1.978 to 0.3082	-1.072 to 1.367	-1.072 to 0.8282	-1.756 to 1.580
t=0.2430 df=3	t=0.2919 df=3	t=1.719 df=3	t=0.2841 df=3	t=0.3021 df=3	t=0.1238 df=3
0.8237	0.7894	0.1842	0.7948	0.7823	0.9093
0.422	0.644	-3.34	0.589	-0.488	-0.351
0.4698		0.108		0.4839	

S-mmu-miR-539-4378103	T-mmu-miR-539-4378103	S-mmu-miR-532-3p-4395466	T-mmu-miR-532-3p-4395466	S-mmu-miR-497-4381046	T-mmu-miR-497-4381046
4	4	4	4	4	4
-0.035	-0.251	-0.665	-0.727	-1.872	-3.043
-0.02875	-0.1757	-0.6477	-0.6055	-1.46	-2.297
0.0415	0.2205	-0.356	-0.1375	-0.066	-0.007
0.2003	0.5898	0.6273	0.9598	0.5797	0.7285
0.236	0.656	0.875	1.291	0.743	0.957
**0.071**	**0.2115**	**-0.1255**	**0.07225**	**-0.3153**	**-0.525**
0.1232	0.3957	0.7103	0.8629	1.113	1.74
0.06158	0.1979	0.3551	0.4314	0.5565	0.8698
-0.0739	-0.2541	-0.9612	-0.9429	-1.625	-2.572
0.2159	0.6771	0.7102	1.087	0.9942	1.522
0	0	0	0	0	0
0.071	0.2115	-0.1255	0.07225	-0.3153	-0.525
-0.071	-0.2115	0.1255	-0.07225	0.3153	0.525
-0.07390 to 0.2159	-0.2541 to 0.6771	-0.9612 to 0.7102	-0.9429 to 1.087	-1.625 to 0.9942	-2.572 to 1.522
t=1.153 df=3	t=1.069 df=3	t=0.3534 df=3	t=0.1675 df=3	t=0.5665 df=3	t=0.6036 df=3
0.3325	0.3635	0.7472	0.8777	0.6107	0.5887
0.284	0.846	-0.502	0.289	-1.261	-2.1
0.2615		0.3678		0.4229	

S-mmu-miR-495-4381078	T-mmu-miR-495-4381078	S-mmu-miR-494-4395476	T-mmu-miR-494-4395476	S-mmu-miR-487b-4378102	T-mmu-miR-487b-4378102
4	4	4	4	4	4
-0.25	0.194	-1.309	-2.719	-0.149	0.079
-0.09575	0.2057	-1.204	-2.396	-0.1315	0.08875
0.5245	0.244	-0.049	-1.008	-0.029	0.2135
0.8403	0.448	1.017	1.474	0.09375	0.6773
0.893	0.515	1.092	2.162	0.118	0.8
**0.423**	**0.2993**	**-0.07875**	**-0.6433**	**-0.02225**	**0.3265**
0.498	0.1458	1.196	2.065	0.1167	0.3313
0.249	0.07289	0.5982	1.032	0.05833	0.1656
-0.1629	0.1277	-1.486	-3.073	-0.1595	-0.06325
1.009	0.4708	1.329	1.786	0.115	0.7162
0	0	0	0	0	0
0.423	0.2993	-0.07875	-0.6433	-0.02225	0.3265
-0.423	-0.2993	0.07875	0.6433	0.02225	-0.3265
-0.1629 to 1.009	0.1277 to 0.4708	-1.486 to 1.329	-3.073 to 1.786	-0.1595 to 0.1150	-0.06325 to 0.7162
t=1.699 df=3	t=4.106 df=3	t=0.1317 df=3	t=0.6230 df=3	t=0.3815 df=3	t=1.971 df=3
0.1879	0.0262	0.9036	0.5774	0.7283	0.1433
1.692	1.197	-0.315	-2.573	-0.089	1.306
0.3251		0.3264		0.0471	

S-mmu-miR-486-4378096	T-mmu-miR-486-4378096	S-mmu-miR-484-4381032	T-mmu-miR-484-4381032	S-mmu-miR-449a-4373207	T-mmu-miR-449a-4373207
4	4	4	4	4	4
-0.43	-0.556	-0.667	-0.164	-1.141	-0.663
-0.3905	-0.509	-0.5305	-0.139	-0.556	-0.2068
0.255	0.0435	-0.095	-0.007	1.54	1.999
1.25	1.159	0.4125	0.6965	1.94	3.321
1.406	1.393	0.573	0.912	1.959	3.483
**0.3715**	**0.231**	**-0.071**	**0.1835**	**0.9745**	**1.705**
0.8747	0.8904	0.5074	0.4935	1.451	1.857
0.4373	0.4452	0.2537	0.2467	0.7255	0.9284
-0.6576	-0.8166	-0.668	-0.3971	-0.7327	-0.48
1.401	1.279	0.526	0.7641	2.682	3.889
0	0	0	0	0	0
0.3715	0.231	-0.071	0.1835	0.9745	1.705
-0.3715	-0.231	0.071	-0.1835	-0.9745	-1.705
-0.6576 to 1.401	-0.8166 to 1.279	-0.6680 to 0.5260	-0.3971 to 0.7641	-0.7327 to 2.682	-0.4800 to 3.889
t=0.8495 df=3	t=0.5189 df=3	t=0.2798 df=3	t=0.7437 df=3	t=1.343 df=3	t=1.836 df=3
0.458	0.6397	0.7978	0.511	0.2718	0.1637
1.486	0.924	-0.284	0.734	3.898	6.818
0.4147		0.2496		0.2792	

S-mmu-miR-434-3p-4395734	T-mmu-miR-434-3p-4395734	S-mmu-miR-433-4373205	T-mmu-miR-433-4373205	S-mmu-miR-431-4395173	T-mmu-miR-431-4395173
4	4	4	4	4	4
-0.388	-0.376	-0.222	-0.542	0.305	-0.882
-0.3605	-0.369	-0.18	-0.4858	0.3195	-0.6918
0.003	-0.138	-0.039	-0.0035	0.5645	0.146
0.3013	0.6293	0.3975	0.4968	1.11	1.125
0.307	0.815	0.538	0.559	1.225	1.362
**-0.01875**	**0.04075**	**0.0595**	**0.0025**	**0.6648**	**0.193**
0.3658	0.5554	0.3307	0.5173	0.4261	0.9433
0.1829	0.2777	0.1653	0.2587	0.213	0.4716
-0.4491	-0.6126	-0.3296	-0.6061	0.1635	-0.9167
0.4116	0.6941	0.4486	0.6111	1.166	1.303
0	0	0	0	0	0
-0.01875	0.04075	0.0595	0.0025	0.6648	0.193
0.01875	-0.04075	-0.0595	-0.0025	-0.6648	-0.193
-0.4491 to 0.4116	-0.6126 to 0.6941	-0.3296 to 0.4486	-0.6061 to 0.6111	0.1635 to 1.166	-0.9167 to 1.303
t=0.1025 df=3	t=0.1468 df=3	t=0.3599 df=3	t=0.009665 df=3	t=3.120 df=3	t=0.4092 df=3
0.9248	0.8926	0.7428	0.9929	0.0525	0.7098
-0.075	0.163	0.238	0.009999	2.659	0.772
0.4319		0.4294		0.1986	

S-mmu-miR-425-4380926	T-mmu-miR-425-4380926	S-mmu-miR-411-4381013	T-mmu-miR-411-4381013	S-mmu-miR-410-4378093	T-mmu-miR-410-4378093
4	4	4	4	4	4
-0.463	0.354	-0.514	-0.213	-0.168	-0.688
-0.314	0.379	-0.3585	-0.1735	-0.1625	-0.498
0.234	0.6225	0.117	0.038	-0.1075	0.082
0.4138	0.8683	0.3285	0.2495	0.291	0.4228
0.44	0.894	0.396	0.289	0.411	0.533
**0.1112**	**0.6233**	**0.029**	**0.038**	**0.007**	**0.00225**
0.4035	0.2599	0.3852	0.2186	0.2727	0.507
0.2017	0.1299	0.1926	0.1093	0.1363	0.2535
-0.3634	0.3175	-0.4242	-0.2191	-0.3138	-0.5942
0.5859	0.929	0.4822	0.2951	0.3278	0.5987
0	0	0	0	0	0
0.1112	0.6233	0.029	0.038	0.007	0.00225
-0.1112	-0.6233	-0.029	-0.038	-0.007	-0.00225
-0.3634 to 0.5859	0.3175 to 0.9290	-0.4242 to 0.4822	-0.2191 to 0.2951	-0.3138 to 0.3278	-0.5942 to 0.5987
t=0.5515 df=3	t=4.797 df=3	t=0.1506 df=3	t=0.3477 df=3	t=0.05135 df=3	t=0.008877 df=3
0.6197	0.0172	0.8899	0.751	0.9623	0.9935
0.445	2.493	0.116	0.152	0.028	0.009
0.0384		0.4845		0.4937	

S-mmu-miR-409-3p-4395443	T-mmu-miR-409-3p-4395443	S-mmu-miR-384-5p-4395732	T-mmu-miR-384-5p-4395732	mmu-miR-383-4381093	mmu-miR-383-4381093
4	4	4	4	4	4
-0.131	-0.245	-0.525	-0.319	-0.818	-0.793
-0.036	-0.1568	-0.5045	-0.3018	-0.7145	-0.5947
0.3185	0.1095	-0.197	-0.083	-0.2585	0.199
0.4923	0.5183	0.238	0.4223	-0.1123	0.428
0.527	0.654	0.301	0.535	-0.112	0.438
**0.2583**	**0.157**	**-0.1545**	**0.0125**	**-0.3617**	**0.01075**
0.2832	0.3711	0.3956	0.3903	0.3338	0.5711
0.1416	0.1855	0.1978	0.1951	0.1669	0.2856
-0.07497	-0.2796	-0.6199	-0.4466	-0.7544	-0.6612
0.5915	0.5936	0.3109	0.4716	0.03093	0.6827
0	0	0	0	0	0
0.2583	0.157	-0.1545	0.0125	-0.3617	0.01075
-0.2583	-0.157	0.1545	-0.0125	0.3617	-0.01075
-0.07497 to 0.5915	-0.2796 to 0.5936	-0.6199 to 0.3109	-0.4466 to 0.4716	-0.7544 to 0.03093	-0.6612 to 0.6827
t=1.824 df=3	t=0.8461 df=3	t=0.7812 df=3	t=0.06406 df=3	t=2.168 df=3	t=0.03764 df=3
0.1657	0.4596	0.4917	0.953	0.1187	0.9723
1.033	0.628	-0.618	0.05	-1.447	0.043
0.3398		0.2849		0.1516	

S-mmu-miR-382-4373019	T-mmu-miR-382-4373019	S-mmu-miR-380-5p-4395731	T-mmu-miR-380-5p-4395731	S-mmu-miR-379-4373349	T-mmu-miR-379-4373349
4	4	4	4	4	4
-1.382	-1.998	-0.792	-1.012	-0.347	-0.376
-1.199	-1.688	-0.6535	-0.9953	-0.2562	-0.2612
-0.4745	-0.4015	-0.162	-0.763	0.2005	0.1775
0.772	0.1783	0.4382	0.02875	0.481	0.4663
1.129	0.253	0.613	0.232	0.513	0.531
**-0.3005**	**-0.637**	**-0.1258**	**-0.5765**	**0.1418**	**0.1275**
1.054	1.001	0.5785	0.5713	0.388	0.3826
0.5272	0.5007	0.2892	0.2856	0.194	0.1913
-1.541	-1.815	-0.8063	-1.249	-0.3148	-0.3226
0.94	0.5411	0.5548	0.09563	0.5983	0.5776
0	0	0	0	0	0
-0.3005	-0.637	-0.1258	-0.5765	0.1418	0.1275
0.3005	0.637	0.1258	0.5765	-0.1418	-0.1275
-1.541 to 0.9400	-1.815 to 0.5411	-0.8063 to 0.5548	-1.249 to 0.09563	-0.3148 to 0.5983	-0.3226 to 0.5776
t=0.5700 df=3	t=1.272 df=3	t=0.4348 df=3	t=2.018 df=3	t=0.7306 df=3	t=0.6665 df=3
0.6086	0.2929	0.6931	0.1369	0.5179	0.5528
-1.202	-2.548	-0.503	-2.306	0.567	0.51
0.3299		0.155		0.48	

S-mmu-miR-376c-4395580	T-mmu-miR-376c-4395580	S-mmu-miR-376a-4373347	T-mmu-miR-376a-4373347	S-mmu-miR-370-4395386	T-mmu-miR-370-4395386
4	4	4	4	4	4
-0.383	-0.597	-0.674	-1.039	-0.987	-0.793
-0.2622	-0.5543	-0.4335	-0.9243	-0.8733	-0.6717
0.416	0.1585	0.3805	0.1405	-0.2715	-0.099
1.081	0.956	0.8622	1.026	0.5507	0.263
1.197	1.027	0.992	1.081	0.738	0.314
**0.4115**	**0.1867**	**0.2697**	**0.08075**	**-0.198**	**-0.1692**
0.6947	0.8175	0.6962	1.049	0.7405	0.4898
0.3474	0.4088	0.3481	0.5244	0.3703	0.2449
-0.4059	-0.7751	-0.5493	-1.153	-1.069	-0.7455
1.229	1.149	1.089	1.315	0.6732	0.407
0	0	0	0	0	0
0.4115	0.1867	0.2697	0.08075	-0.198	-0.1692
-0.4115	-0.1867	-0.2697	-0.08075	0.198	0.1692
-0.4059 to 1.229	-0.7751 to 1.149	-0.5493 to 1.089	-1.153 to 1.315	-1.069 to 0.6732	-0.7455 to 0.4070
t=1.185 df=3	t=0.4569 df=3	t=0.7750 df=3	t=0.1540 df=3	t=0.5348 df=3	t=0.6910 df=3
0.3215	0.6788	0.4948	0.8874	0.6299	0.5392
1.646	0.747	1.079	0.323	-0.792	-0.677
0.3449		0.387		0.4752	

S-mmu-miR-365-4373194	T-mmu-miR-365-4373194	S-mmu-miR-351-4373345	T-mmu-miR-351-4373345	S-mmu-miR-34b-3p-4395748	T-mmu-miR-34b-3p-4395748
4	4	4	4	4	4
-0.055	-0.092	-0.313	-1.178	-0.624	0.009
-0.046	0.1333	-0.1745	-0.7925	-0.4283	0.1032
0.2535	0.855	0.2935	0.632	0.3885	0.4495
2.138	1.731	1.422	1.151	0.7688	1.067
2.675	2.008	1.781	1.235	0.819	1.251
**0.7817**	**0.9065**	**0.5138**	**0.3303**	**0.243**	**0.5398**
1.29	0.8602	0.8929	1.068	0.6406	0.5202
0.6449	0.4301	0.4465	0.5338	0.3203	0.2601
-0.7358	-0.1055	-0.5368	-0.9258	-0.5107	-0.0723
2.299	1.919	1.564	1.586	0.9967	1.152
0	0	0	0	0	0
0.7817	0.9065	0.5138	0.3303	0.243	0.5398
-0.7817	-0.9065	-0.5138	-0.3303	-0.243	-0.5398
-0.7358 to 2.299	-0.1055 to 1.919	-0.5368 to 1.564	-0.9258 to 1.586	-0.5107 to 0.9967	-0.07230 to 1.152
t=1.212 df=3	t=2.108 df=3	t=1.151 df=3	t=0.6187 df=3	t=0.7587 df=3	t=2.075 df=3
0.3122	0.1256	0.3333	0.5799	0.5032	0.1296
3.127	3.626	2.055	1.321	0.972	2.159
0.4387		0.4004		0.2495	

S-mmu-miR-34a-4395168	T-mmu-miR-34a-4395168	S-mmu-miR-342-3p-4395371	T-mmu-miR-342-3p-4395371	S-mmu-miR-340-5p-4395369	T-mmu-miR-340-5p-4395369
4	4	4	4	4	4
-1.497	-1.06	-0.188	0.045	0.05	0.385
-1.36	-0.944	-0.09375	0.05375	0.173	0.406
0.0485	0.242	0.3005	0.2325	0.7755	0.8065
2.279	1.984	0.4233	0.5635	1.32	1.37
2.691	2.285	0.427	0.623	1.423	1.445
**0.3228**	**0.4273**	**0.21**	**0.2833**	**0.756**	**0.8608**
1.919	1.542	0.2868	0.2732	0.5925	0.5168
0.9597	0.7712	0.1434	0.1366	0.2962	0.2584
-1.935	-1.387	-0.1274	-0.03813	0.05894	0.2527
2.581	2.242	0.5474	0.6046	1.453	1.469
0	0	0	0	0	0
0.3228	0.4273	0.21	0.2833	0.756	0.8608
-0.3228	-0.4273	-0.21	-0.2833	-0.756	-0.8608
-1.935 to 2.581	-1.387 to 2.242	-0.1274 to 0.5474	-0.03813 to 0.6046	0.05894 to 1.453	0.2527 to 1.469
t=0.3363 df=3	t=0.5540 df=3	t=1.465 df=3	t=2.074 df=3	t=2.552 df=3	t=3.331 df=3
0.7588	0.6182	0.2393	0.1298	0.0838	0.0447
1.291	1.709	0.84	1.133	3.024	3.443
0.4676		0.3621		0.3994	

S-mmu-miR-340-3p-4395370	T-mmu-miR-340-3p-4395370	S-mmu-miR-339-5p-4395368	T-mmu-miR-339-5p-4395368	S-mmu-miR-338-3p-4395363	T-mmu-miR-338-3p-4395363
4	4	4	4	4	4
-1.058	-0.478	-0.11	-0.861	-0.27	-2.687
-0.8578	-0.4773	-0.094	-0.7	-0.09075	-2
-0.202	-0.155	0.232	0.019	0.4665	0.1705
0.9825	1.338	0.9217	2.137	1.237	0.3898
1.359	1.729	1.059	2.764	1.487	0.427
**-0.02575**	**0.2352**	**0.3533**	**0.4853**	**0.5375**	**-0.4798**
1.009	1.041	0.5467	1.587	0.7221	1.479
0.5043	0.5204	0.2734	0.7933	0.3611	0.7395
-1.212	-0.9892	-0.2899	-1.381	-0.3121	-2.22
1.161	1.46	0.9964	2.352	1.387	1.26
0	0	0	0	0	0
-0.02575	0.2352	0.3533	0.4853	0.5375	-0.4798
0.02575	-0.2352	-0.3533	-0.4853	-0.5375	0.4798
-1.212 to 1.161	-0.9892 to 1.460	-0.2899 to 0.9964	-1.381 to 2.352	-0.3121 to 1.387	-2.220 to 1.260
t=0.05107 df=3	t=0.4521 df=3	t=1.292 df=3	t=0.6117 df=3	t=1.489 df=3	t=0.6487 df=3
0.9625	0.6819	0.2868	0.584	0.2333	0.5628
-0.103	0.941	1.413	1.941	2.15	-1.919
0.3655		0.4401		0.1313	

S-mmu-miR-337-5p-4395645	T-mmu-miR-337-5p-4395645	S-mmu-miR-337-3p-4395662	T-mmu-miR-337-3p-4395662	S-mmu-miR-335-3p-4395296	T-mmu-miR-335-3p-4395296
4	4	4	4	4	4
-0.906	-0.33	-3.979	-4.87	-0.623	-1.054
-0.8018	-0.2745	-3.062	-4.118	-0.4972	-0.859
-0.0095	0.2095	0.607	-0.222	-0.1015	-0.169
0.4858	1.058	4.91	4.708	0.433	1.3
0.491	1.235	6.038	5.805	0.605	1.754
**-0.1085**	**0.331**	**0.8183**	**0.1228**	**-0.05525**	**0.0905**
0.7012	0.7036	4.165	4.576	0.5044	1.188
0.3506	0.3518	2.082	2.288	0.2522	0.594
-0.9334	-0.4968	-4.082	-5.261	-0.6487	-1.307
0.7164	1.159	5.718	5.507	0.5382	1.488
0	0	0	0	0	0
-0.1085	0.331	0.8183	0.1228	-0.05525	0.0905
0.1085	-0.331	-0.8183	-0.1228	0.05525	-0.0905
-0.9334 to 0.7164	-0.4968 to 1.159	-4.082 to 5.718	-5.261 to 5.507	-0.6487 to 0.5382	-1.307 to 1.488
t=0.3095 df=3	t=0.9408 df=3	t=0.3929 df=3	t=0.05365 df=3	t=0.2191 df=3	t=0.1524 df=3
0.7772	0.4162	0.7206	0.9606	0.8406	0.8886
-0.434	1.324	3.273	0.491	-0.221	0.362
0.2051		0.4148		0.4144	

S-mmu-miR-331-3p-4373046	T-mmu-miR-331-3p-4373046	S-mmu-miR-328-4373049	T-mmu-miR-328-4373049	S-mmu-miR-323-3p-4395338	T-mmu-miR-323-3p-4395338
4	4	4	4	4	4
-0.19	-0.084	-0.697	-0.537	-0.311	0.105
-0.1883	-0.051	-0.6145	-0.3932	-0.2347	0.107
0.193	0.3095	-0.196	0.3005	0.0895	0.147
0.818	0.9408	0.5292	0.593	0.419	0.3025
0.901	1.064	0.714	0.603	0.497	0.343
**0.2743**	**0.3998**	**-0.09375**	**0.1668**	**0.09125**	**0.1855**
0.549	0.5255	0.6044	0.5352	0.339	0.1104
0.2745	0.2627	0.3022	0.2676	0.1695	0.0552
-0.3717	-0.2184	-0.8048	-0.4629	-0.3075	0.05562
0.9202	1.018	0.6173	0.7964	0.49	0.3154
0	0	0	0	0	0
0.2743	0.3998	-0.09375	0.1668	0.09125	0.1855
-0.2743	-0.3998	0.09375	-0.1668	-0.09125	-0.1855
-0.3717 to 0.9202	-0.2184 to 1.018	-0.8048 to 0.6173	-0.4629 to 0.7964	-0.3075 to 0.4900	0.05562 to 0.3154
t=0.9990 df=3	t=1.522 df=3	t=0.3102 df=3	t=0.6232 df=3	t=0.5384 df=3	t=3.361 df=3
0.3914	0.2255	0.7767	0.5773	0.6277	0.0437
1.097	1.599	-0.375	0.667	0.365	0.742
0.3762		0.2713		0.308	

S-mmu-miR-320-4395388	T-mmu-miR-320-4395388	S-mmu-miR-31-4373331	T-mmu-miR-31-4373331	S-mmu-miR-30e-4395334	T-mmu-miR-30e-4395334
4	4	4	4	4	4
-0.185	-0.564	-0.111	-0.678	-0.239	0.028
-0.0765	-0.3395	-0.05525	-0.4723	-0.1828	0.04175
0.3345	0.3565	0.195	0.2175	0.0605	0.149
0.5183	0.514	0.617	0.5465	0.1808	0.5143
0.551	0.559	0.73	0.632	0.196	0.614
**0.2588**	**0.177**	**0.2523**	**0.09725**	**0.0195**	**0.235**
0.3206	0.5035	0.3562	0.5557	0.1936	0.2646
0.1603	0.2517	0.1781	0.2778	0.0968	0.1323
-0.1185	-0.4153	-0.1668	-0.5565	-0.2083	-0.07627
0.636	0.7693	0.6713	0.751	0.2473	0.5463
0	0	0	0	0	0
0.2588	0.177	0.2523	0.09725	0.0195	0.235
-0.2588	-0.177	-0.2523	-0.09725	-0.0195	-0.235
-0.1185 to 0.6360	-0.4153 to 0.7693	-0.1668 to 0.6713	-0.5565 to 0.7510	-0.2083 to 0.2473	-0.07627 to 0.5463
t=1.614 df=3	t=0.7031 df=3	t=1.417 df=3	t=0.3500 df=3	t=0.2015 df=3	t=1.776 df=3
0.2049	0.5326	0.2516	0.7495	0.8532	0.1737
1.035	0.708	1.009	0.389	0.078	0.94
0.3967		0.3276		0.1183	

S-mmu-miR-30d-4373059	T-mmu-miR-30d-4373059	S-mmu-miR-30c-4373060	T-mmu-miR-30c-4373060	S-mmu-miR-30b-4373290	T-mmu-miR-30b-4373290
4	4	4	4	4	4
-0.194	-0.8	-0.595	-0.288	-0.445	-0.512
-0.153	-0.6068	-0.4883	-0.1793	-0.415	-0.349
0.2235	0.0585	0.0445	0.346	-0.007	0.3335
0.4777	0.4718	0.3088	0.6673	0.4183	0.7565
0.478	0.581	0.326	0.708	0.454	0.833
**0.1828**	**-0.0255**	**-0.045**	**0.278**	**-0.00125**	**0.247**
0.3469	0.5763	0.4268	0.4449	0.4496	0.58
0.1734	0.2882	0.2134	0.2224	0.2248	0.29
-0.2253	-0.7035	-0.5471	-0.2454	-0.5302	-0.4354
0.5908	0.6525	0.4571	0.8014	0.5277	0.9294
0	0	0	0	0	0
0.1828	-0.0255	-0.045	0.278	-0.00125	0.247
-0.1828	0.0255	0.045	-0.278	0.00125	-0.247
-0.2253 to 0.5908	-0.7035 to 0.6525	-0.5471 to 0.4571	-0.2454 to 0.8014	-0.5302 to 0.5277	-0.4354 to 0.9294
t=1.054 df=3	t=0.08849 df=3	t=0.2109 df=3	t=1.250 df=3	t=0.005560 df=3	t=0.8517 df=3
0.3694	0.9351	0.8465	0.3	0.9959	0.457
0.731	-0.102	-0.18	1.112	-0.005	0.988
0.2793		0.1675		0.262	

S-mmu-miR-30a-4373061	T-mmu-miR-30a-4373061	S-mmu-miR-301b-4395730	T-mmu-miR-301b-4395730	S-mmu-miR-301a-4373064	T-mmu-miR-301a-4373064
4	4	4	4	4	4
-0.481	-0.301	-0.283	-1.038	-1.029	-0.668
-0.456	-0.2265	-0.1705	-0.8803	-0.8797	-0.4135
-0.096	0.044	0.1985	-0.0865	-0.042	0.365
0.2745	0.355	0.6935	0.8183	1.102	0.4137
0.303	0.443	0.848	1.013	1.353	0.425
**-0.0925**	**0.0575**	**0.2405**	**-0.0495**	**0.06**	**0.1218**
0.3957	0.3065	0.465	0.8783	1.03	0.5274
0.1979	0.1533	0.2325	0.4391	0.515	0.2637
-0.5581	-0.3032	-0.3065	-1.083	-1.152	-0.4987
0.3731	0.4182	0.7875	0.9838	1.272	0.7422
0	0	0	0	0	0
-0.0925	0.0575	0.2405	-0.0495	0.06	0.1218
0.0925	-0.0575	-0.2405	0.0495	-0.06	-0.1218
-0.5581 to 0.3731	-0.3032 to 0.4182	-0.3065 to 0.7875	-1.083 to 0.9838	-1.152 to 1.272	-0.4987 to 0.7422
t=0.4675 df=3	t=0.3751 df=3	t=1.034 df=3	t=0.1127 df=3	t=0.1165 df=3	t=0.4617 df=3
0.672	0.7325	0.377	0.9174	0.9146	0.6757
-0.37	0.23	0.962	-0.198	0.24	0.487
0.2854		0.2904		0.4592	

S-mmu-miR-29c-4395171	T-mmu-miR-29c-4395171	S-mmu-miR-29a-4395223	T-mmu-miR-29a-4395223	mmu-miR-28-4373067	mmu-miR-28-4373067
4	4	4	4	4	4
-0.538	-0.8	-0.12	0.069	0.075	-0.113
-0.454	-0.6937	-0.06875	0.1113	0.088	0.07
0.1025	0.231	0.203	0.361	0.1935	0.736
0.8593	0.849	0.5723	0.652	1.099	1.629
1.01	0.853	0.656	0.708	1.379	1.888
**0.1693**	**0.1288**	**0.2355**	**0.3748**	**0.4602**	**0.8118**
0.6835	0.8451	0.3332	0.28	0.6174	0.8271
0.3417	0.4225	0.1666	0.14	0.3087	0.4136
-0.6349	-0.8655	-0.1566	0.04535	-0.2662	-0.1613
0.9734	1.123	0.6276	0.7042	1.187	1.785
0	0	0	0	0	0
0.1693	0.1288	0.2355	0.3748	0.4602	0.8118
-0.1693	-0.1288	-0.2355	-0.3748	-0.4602	-0.8118
-0.6349 to 0.9734	-0.8655 to 1.123	-0.1566 to 0.6276	0.04535 to 0.7042	-0.2662 to 1.187	-0.1613 to 1.785
t=0.4953 df=3	t=0.3047 df=3	t=1.413 df=3	t=2.677 df=3	t=1.491 df=3	t=1.963 df=3
0.6544	0.7805	0.2524	0.0752	0.2328	0.1444
0.677	0.515	0.942	1.499	1.841	3.247
0.4715		0.273		0.2606	

mmu-miR-27b-4373068	mmu-miR-27b-4373068	S-mmu-miR-27a-4373287	T-mmu-miR-27a-4373287	S-mmu-miR-26b-4395167	T-mmu-miR-26b-4395167
4	4	4	4	4	4
-0.978	-2.357	-0.208	-1.636	-0.168	-0.642
-0.8175	-1.902	-0.1283	-1.193	-0.142	-0.4565
0.051	-0.3295	0.243	0.184	0.295	0.4105
1.327	0.6048	0.951	1.453	1.131	1.27
1.623	0.847	1.143	1.86	1.29	1.453
**0.1868**	**-0.5423**	**0.3553**	**0.148**	**0.428**	**0.408**
1.119	1.342	0.5767	1.428	0.681	0.8921
0.5595	0.6708	0.2884	0.7142	0.3405	0.446
-1.13	-2.121	-0.3233	-1.533	-0.3732	-0.6415
1.503	1.036	1.034	1.829	1.229	1.458
0	0	0	0	0	0
0.1868	-0.5423	0.3553	0.148	0.428	0.408
-0.1868	0.5423	-0.3553	-0.148	-0.428	-0.408
-1.130 to 1.503	-2.121 to 1.036	-0.3233 to 1.034	-1.533 to 1.829	-0.3732 to 1.229	-0.6415 to 1.458
t=0.3338 df=3	t=0.8083 df=3	t=1.232 df=3	t=0.2072 df=3	t=1.257 df=3	t=0.9147 df=3
0.7605	0.478	0.3057	0.8491	0.2977	0.4278
0.747	-2.169	1.421	0.592	1.712	1.632
0.218		0.3984		0.4864	

S-mmu-miR-26a-4395166	T-mmu-miR-26a-4395166	S-mmu-miR-24-4373072	T-mmu-miR-24-4373072	S-mmu-miR-23b-4373073	T-mmu-miR-23b-4373073
4	4	4	4	4	4
-0.031	-0.275	-0.242	0.182	-0.395	-0.114
0.0045	-0.245	-0.1472	0.234	-0.3125	-0.01325
0.3355	0.105	0.159	0.403	0.3115	0.451
0.563	0.5008	0.2358	0.5285	0.7458	1.164
0.564	0.546	0.254	0.566	0.765	1.348
**0.301**	**0.1203**	**0.0825**	**0.3885**	**0.2483**	**0.534**
0.3069	0.3971	0.2216	0.158	0.5693	0.6188
0.1535	0.1986	0.1108	0.07901	0.2846	0.3094
-0.06007	-0.347	-0.1783	0.2026	-0.4215	-0.194
0.6621	0.5875	0.3433	0.5744	0.918	1.262
0	0	0	0	0	0
0.301	0.1203	0.0825	0.3885	0.2483	0.534
-0.301	-0.1203	-0.0825	-0.3885	-0.2483	-0.534
-0.06007 to 0.6621	-0.3470 to 0.5875	-0.1783 to 0.3433	0.2026 to 0.5744	-0.4215 to 0.9180	-0.1940 to 1.262
t=1.962 df=3	t=0.6056 df=3	t=0.7444 df=3	t=4.917 df=3	t=0.8721 df=3	t=1.726 df=3
0.1446	0.5875	0.5106	0.0161	0.4473	0.1828
1.204	0.481	0.33	1.554	0.993	2.136
0.2492		0.0328		0.261	

S-mmu-miR-223-4395406	T-mmu-miR-223-4395406	S-mmu-miR-222-4395387	T-mmu-miR-222-4395387	S-mmu-miR-218-4373081	T-mmu-miR-218-4373081
4	4	4	4	4	4
-0.092	0.002	0.432	0.584	-0.185	-0.333
-0.06675	0.05	0.434	0.594	-0.175	-0.2543
0.1725	0.4015	0.5505	0.647	0.2725	0.2475
0.9855	1.604	0.6633	0.79	0.7088	0.9068
1.202	1.935	0.664	0.83	0.715	1.038
**0.3638**	**0.685**	**0.5493**	**0.677**	**0.2688**	**0.3**
0.5879	0.871	0.1308	0.1079	0.5012	0.6033
0.294	0.4355	0.06541	0.05394	0.2506	0.3016
-0.328	-0.3397	0.3953	0.5501	-0.3209	-0.4098
1.055	1.71	0.7032	0.8039	0.8584	1.01
0	0	0	0	0	0
0.3638	0.685	0.5493	0.677	0.2688	0.3
-0.3638	-0.685	-0.5493	-0.677	-0.2688	-0.3
-0.3280 to 1.055	-0.3397 to 1.710	0.3953 to 0.7032	0.5501 to 0.8039	-0.3209 to 0.8584	-0.4098 to 1.010
t=1.237 df=3	t=1.573 df=3	t=8.397 df=3	t=12.55 df=3	t=1.072 df=3	t=0.9946 df=3
0.304	0.2138	0.0035	0.0011	0.3621	0.3933
1.455	2.74	2.197	2.708	1.075	1.2
0.2817		0.0913		0.4695	

S-mmu-miR-217-4395686	T-mmu-miR-217-4395686	S-mmu-miR-214-4395417	T-mmu-miR-214-4395417	S-mmu-miR-210-4373089	T-mmu-miR-210-4373089
4	4	4	4	4	4
-2.656	-0.992	-0.348	0.07	-0.542	-0.741
-2.196	-0.8665	-0.2488	0.2005	-0.468	-0.6982
-0.7115	-0.25	0.2245	0.6705	-0.135	-0.0625
-0.2508	0.371	0.5365	0.9688	0.4643	0.6048
-0.132	0.498	0.582	1.042	0.627	0.658
**-1.053**	**-0.2485**	**0.1707**	**0.6133**	**-0.04625**	**-0.052**
1.106	0.6391	0.4105	0.4074	0.4965	0.7057
0.5532	0.3195	0.2053	0.2037	0.2482	0.3529
-2.355	-1	-0.3122	0.134	-0.6303	-0.8823
0.249	0.5034	0.6537	1.093	0.5378	0.7783
0	0	0	0	0	0
-1.053	-0.2485	0.1707	0.6133	-0.04625	-0.052
1.053	0.2485	-0.1707	-0.6133	0.04625	0.052
-2.355 to 0.2490	-1.000 to 0.5034	-0.3122 to 0.6537	0.1340 to 1.093	-0.6303 to 0.5378	-0.8823 to 0.7783
t=1.903 df=3	t=0.7777 df=3	t=0.8318 df=3	t=3.011 df=3	t=0.1863 df=3	t=0.1474 df=3
0.1532	0.4935	0.4665	0.0572	0.8641	0.8922
-4.211	-0.994	0.683	2.453	-0.185	-0.208
0.1274		0.0884		0.4949	

S-mmu-miR-20b-4373263	T-mmu-miR-20b-4373263	S-mmu-miR-20a-4373286	T-mmu-miR-20a-4373286	S-mmu-miR-204-4373094	T-mmu-miR-204-4373094
4	4	4	4	4	4
-3.305	-0.671	-0.168	-1.825	-0.598	-0.388
-2.589	-0.5933	-0.1093	-1.414	-0.5313	-0.3323
-0.339	0.198	0.087	0.268	0.058	0.051
0.56	1.092	0.566	1.064	0.474	0.7898
0.826	1.204	0.719	1.18	0.483	0.964
**-0.7892**	**0.2323**	**0.1812**	**-0.02725**	**0.00025**	**0.1695**
1.767	0.8917	0.3785	1.325	0.5478	0.5954
0.8834	0.4458	0.1892	0.6625	0.2739	0.2977
-2.868	-0.8168	-0.264	-1.586	-0.6442	-0.531
1.289	1.281	0.6265	1.532	0.6447	0.87
0	0	0	0	0	0
-0.7892	0.2323	0.1812	-0.02725	0.00025	0.1695
0.7892	-0.2323	-0.1812	0.02725	-0.00025	-0.1695
-2.868 to 1.289	-0.8168 to 1.281	-0.2640 to 0.6265	-1.586 to 1.532	-0.6442 to 0.6447	-0.5310 to 0.8700
t=0.8935 df=3	t=0.5209 df=3	t=0.9578 df=3	t=0.04113 df=3	t=0.0009127 df=3	t=0.5694 df=3
0.4374	0.6384	0.4088	0.9698	0.9993	0.6089
-3.157	0.929	0.725	-0.109	0.001	0.678
0.1709		0.3862		0.3451	

S-mmu-miR-203-4373095	T-mmu-miR-203-4373095	S-mmu-miR-200c-4395411	T-mmu-miR-200c-4395411	S-mmu-miR-19b-4373098	T-mmu-miR-19b-4373098
4	4	4	4	4	4
-0.564	-0.363	0.253	-0.295	0.115	-0.39
-0.3725	-0.1742	0.3115	-0.1173	0.1375	-0.2725
0.269	0.5135	0.608	0.478	0.2515	0.2985
0.609	0.7243	0.8618	0.6007	0.3003	0.6055
0.7	0.754	0.906	0.621	0.301	0.635
**0.1685**	**0.3545**	**0.5938**	**0.3205**	**0.2298**	**0.2105**
0.5317	0.5015	0.2848	0.4189	0.08853	0.4661
0.2659	0.2507	0.1424	0.2095	0.04427	0.2331
-0.4571	-0.2355	0.2587	-0.1723	0.1256	-0.3379
0.7941	0.9445	0.9288	0.8133	0.3339	0.7589
0	0	0	0	0	0
0.1685	0.3545	0.5938	0.3205	0.2298	0.2105
-0.1685	-0.3545	-0.5938	-0.3205	-0.2298	-0.2105
-0.4571 to 0.7941	-0.2355 to 0.9445	0.2587 to 0.9288	-0.1723 to 0.8133	0.1256 to 0.3339	-0.3379 to 0.7589
t=0.6338 df=3	t=1.414 df=3	t=4.170 df=3	t=1.530 df=3	t=5.190 df=3	t=0.9032 df=3
0.5712	0.2523	0.0251	0.2235	0.0139	0.433
0.674	1.418	2.375	1.282	0.919	0.842
0.3145		0.161		0.469	

S-mmu-miR-19a-4373099	T-mmu-miR-19a-4373099	S-mmu-miR-199a-3p-4395415	T-mmu-miR-199a-3p-4395415	S-mmu-miR-196b-4395326	T-mmu-miR-196b-4395326
4	4	4	4	4	4
0.189	-0.869	-0.911	-0.255	-0.545	0.093
0.309	-0.4268	-0.7248	-0.1828	-0.4688	0.2133
0.7665	1.003	0.013	0.132	0.058	0.5985
1.106	1.146	0.4178	0.5495	0.5795	0.7355
1.186	1.159	0.493	0.656	0.654	0.773
**0.727**	**0.574**	**-0.098**	**0.1663**	**0.05625**	**0.5157**
0.4172	0.9685	0.6052	0.3825	0.5466	0.2943
0.2086	0.4842	0.3026	0.1912	0.2733	0.1471
0.2361	-0.5654	-0.8101	-0.2837	-0.5869	0.1695
1.218	1.713	0.6141	0.6162	0.6994	0.862
0	0	0	0	0	0
0.727	0.574	-0.098	0.1663	0.05625	0.5157
-0.727	-0.574	0.098	-0.1663	-0.05625	-0.5157
0.2361 to 1.218	-0.5654 to 1.713	-0.8101 to 0.6141	-0.2837 to 0.6162	-0.5869 to 0.6994	0.1695 to 0.8620
t=3.485 df=3	t=1.185 df=3	t=0.3238 df=3	t=0.8693 df=3	t=0.2058 df=3	t=3.505 df=3
0.0399	0.3212	0.7673	0.4486	0.8501	0.0393
2.908	2.296	-0.392	0.665	0.225	2.063
0.3907		0.2441		0.0946	

S-mmu-miR-195-4373105	T-mmu-miR-195-4373105	S-mmu-miR-192-4373108	T-mmu-miR-192-4373108	S-mmu-miR-187-4373307	T-mmu-miR-187-4373307
4	4	4	4	4	4
0	0.402	-0.857	-1.176	-0.201	-1.345
0.00575	0.4145	-0.835	-0.861	-0.1053	-0.9173
0.1515	0.459	-0.2145	0.262	0.39	0.7985
0.3003	0.4743	0.3955	0.8488	1.236	1.251
0.307	0.477	0.414	0.985	1.448	1.257
**0.1525**	**0.4493**	**-0.218**	**0.08325**	**0.5067**	**0.3772**
0.1635	0.03312	0.6886	0.9176	0.7073	1.221
0.08173	0.01656	0.3443	0.4588	0.3536	0.6103
-0.03981	0.4103	-1.028	-0.9963	-0.3253	-1.059
0.3448	0.4882	0.5922	1.163	1.339	1.813
0	0	0	0	0	0
0.1525	0.4493	-0.218	0.08325	0.5067	0.3772
-0.1525	-0.4493	0.218	-0.08325	-0.5067	-0.3772
-0.03981 to 0.3448	0.4103 to 0.4882	-1.028 to 0.5922	-0.9963 to 1.163	-0.3253 to 1.339	-1.059 to 1.813
t=1.866 df=3	t=27.13 df=3	t=0.6331 df=3	t=0.1814 df=3	t=1.433 df=3	t=0.6182 df=3
0.1589	0.0001	0.5716	0.8676	0.2473	0.5802
0.61	1.797	-0.872	0.333	2.027	1.509
0.006		0.3092		0.4302	

S-mmu-miR-186-4395396	T-mmu-miR-186-4395396	S-mmu-miR-185-4395382	T-mmu-miR-185-4395382	S-mmu-miR-184-4373113	T-mmu-miR-184-4373113
4	4	4	4	4	4
-0.072	-0.53	-0.974	-0.564	-0.238	-1.062
-0.058	-0.3375	-0.9413	-0.1063	-0.1447	-0.8975
0.0175	0.4075	0.5765	1.271	0.645	-0.2465
0.2543	0.596	2.608	2.973	1.208	1.103
0.322	0.603	2.812	3.539	1.225	1.5
**0.07125**	**0.222**	**0.7478**	**1.379**	**0.5693**	**-0.01375**
0.1746	0.5278	1.942	1.68	0.7333	1.088
0.08728	0.2639	0.971	0.8399	0.3667	0.5438
-0.1341	-0.3989	-1.537	-0.5969	-0.2935	-1.293
0.2766	0.8429	3.033	3.355	1.432	1.266
0	0	0	0	0	0
0.07125	0.222	0.7478	1.379	0.5693	-0.01375
-0.07125	-0.222	-0.7478	-1.379	-0.5693	0.01375
-0.1341 to 0.2766	-0.3989 to 0.8429	-1.537 to 3.033	-0.5969 to 3.355	-0.2935 to 1.432	-1.293 to 1.266
t=0.8163 df=3	t=0.8413 df=3	t=0.7701 df=3	t=1.642 df=3	t=1.552 df=3	t=0.02529 df=3
0.4741	0.462	0.4973	0.1991	0.2184	0.9814
0.285	0.888	2.991	5.517	2.277	-0.055
0.3035		0.3201		0.2041	

S-mmu-miR-183-4395380	T-mmu-miR-183-4395380	S-mmu-miR-182-4395729	T-mmu-miR-182-4395729	S-mmu-miR-181a-4373117	T-mmu-miR-181a-4373117
4	4	4	4	4	4
-0.87	-0.731	-0.867	-0.472	-1.061	-0.602
-0.777	-0.675	-0.7665	-0.4548	-0.7807	-0.3757
0.0855	-0.0855	-0.346	-0.287	0.1285	0.3715
0.8265	0.4223	-0.1475	0.336	0.845	1.106
0.879	0.451	-0.121	0.505	1.061	1.328
**0.045**	**-0.1128**	**-0.42**	**-0.1353**	**0.06425**	**0.3672**
0.8597	0.5935	0.3309	0.4458	0.8713	0.7899
0.4298	0.2968	0.1654	0.2229	0.4356	0.395
-0.9664	-0.811	-0.8093	-0.6598	-0.9608	-0.5621
1.056	0.5855	-0.0307	0.3893	1.089	1.297
0	0	0	0	0	0
0.045	-0.1128	-0.42	-0.1353	0.06425	0.3672
-0.045	0.1128	0.42	0.1353	-0.06425	-0.3672
-0.9664 to 1.056	-0.8110 to 0.5855	-0.8093 to -0.03070	-0.6598 to 0.3893	-0.9608 to 1.089	-0.5621 to 1.297
t=0.1047 df=3	t=0.3799 df=3	t=2.539 df=3	t=0.6067 df=3	t=0.1475 df=3	t=0.9298 df=3
0.9232	0.7293	0.0848	0.5869	0.8921	0.421
0.18	-0.451	-1.68	-0.541	0.257	1.469
0.3864		0.1723		0.3124	

S-mmu-miR-17-4395419	T-mmu-miR-17-4395419	S-mmu-miR-16-4373121	T-mmu-miR-16-4373121	S-mmu-miR-15b-4373122	T-mmu-miR-15b-4373122
4	4	4	4	4	4
0.044	0.329	0.276	0.059	-1.546	-1.848
0.1538	0.359	0.2978	0.1068	-1.406	-1.504
0.5965	0.4695	0.4285	0.2515	-0.919	-0.1445
0.8233	0.6325	0.6545	0.6018	0.5027	0.3147
0.861	0.68	0.708	0.718	0.955	0.359
**0.5245**	**0.487**	**0.4603**	**0.32**	**-0.6073**	**-0.4445**
0.356	0.1457	0.1879	0.2804	1.084	1.001
0.178	0.07284	0.09395	0.1402	0.542	0.5007
0.1056	0.3156	0.2392	-0.00992	-1.882	-1.623
0.9434	0.6584	0.6813	0.6499	0.668	0.7337
0	0	0	0	0	0
0.5245	0.487	0.4603	0.32	-0.6073	-0.4445
-0.5245	-0.487	-0.4603	-0.32	0.6073	0.4445
0.1056 to 0.9434	0.3156 to 0.6584	0.2392 to 0.6813	-0.009920 to 0.6499	-1.882 to 0.6680	-1.623 to 0.7337
t=2.946 df=3	t=6.686 df=3	t=4.899 df=3	t=2.282 df=3	t=1.120 df=3	t=0.8877 df=3
0.0602	0.0068	0.0163	0.1067	0.3441	0.4401
2.098	1.948	1.841	1.28	-2.429	-1.778
0.4259		0.2189		0.4164	

S-mmu-miR-15a-4373123	T-mmu-miR-15a-4373123	S-mmu-miR-155-4395701	T-mmu-miR-155-4395701	S-mmu-miR-152-4395170	T-mmu-miR-152-4395170
4	4	4	4	4	4
-2.32	-0.328	-0.344	-1.268	-0.063	-0.476
-1.519	-0.237	-0.259	-0.996	-0.01375	-0.36
1.75	1.456	0.0925	0.3595	0.1465	0.26
3.927	3.687	0.2048	0.9365	0.1973	1.257
4.365	3.957	0.21	0.949	0.21	1.499
**1.386**	**1.635**	**0.01275**	**0.1**	**0.11**	**0.3858**
2.85	2.109	0.2566	1.05	0.1196	0.8488
1.425	1.054	0.1283	0.5251	0.0598	0.4244
-1.967	-0.8458	-0.2891	-1.136	-0.0307	-0.6129
4.739	4.116	0.3146	1.336	0.2507	1.384
0	0	0	0	0	0
1.386	1.635	0.01275	0.1	0.11	0.3858
-1.386	-1.635	-0.01275	-0.1	-0.11	-0.3858
-1.967 to 4.739	-0.8458 to 4.116	-0.2891 to 0.3146	-1.136 to 1.336	-0.03070 to 0.2507	-0.6129 to 1.384
t=0.9728 df=3	t=1.551 df=3	t=0.09938 df=3	t=0.1904 df=3	t=1.840 df=3	t=0.9089 df=3
0.4024	0.2188	0.9271	0.8611	0.1631	0.4304
5.545	6.54	0.051	0.4	0.44	1.543
0.4465		0.4385		0.2719	

S-mmu-miR-151-3p-4373304	T-mmu-miR-151-3p-4373304	S-mmu-miR-150-4373127	T-mmu-miR-150-4373127	S-mmu-miR-148a-4373130	T-mmu-miR-148a-4373130
4	4	4	4	4	4
-0.401	-0.257	-0.026	0.214	-0.145	-0.648
-0.3008	-0.069	-0.00075	0.2155	-0.0605	-0.424
0.2265	0.6175	0.122	0.283	0.438	0.721
1.072	1.48	0.3625	0.5013	2.564	2.239
1.278	1.726	0.427	0.553	3.191	2.587
**0.3325**	**0.676**	**0.1613**	**0.3333**	**0.9805**	**0.8452**
0.7204	0.8185	0.1942	0.1586	1.512	1.383
0.3602	0.4093	0.09712	0.07932	0.7562	0.6917
-0.5151	-0.287	-0.06727	0.1466	-0.7988	-0.7824
1.18	1.639	0.3898	0.5199	2.76	2.473
0	0	0	0	0	0
0.3325	0.676	0.1613	0.3333	0.9805	0.8452
-0.3325	-0.676	-0.1613	-0.3333	-0.9805	-0.8452
-0.5151 to 1.180	-0.2870 to 1.639	-0.06727 to 0.3898	0.1466 to 0.5199	-0.7988 to 2.760	-0.7824 to 2.473
t=0.9231 df=3	t=1.652 df=3	t=1.660 df=3	t=4.201 df=3	t=1.297 df=3	t=1.222 df=3
0.4241	0.1971	0.1954	0.0246	0.2855	0.309
1.33	2.704	0.645	1.333	3.922	3.381
0.2759		0.1096		0.4497	

S-mmu-miR-146a-4373132	T-mmu-miR-146a-4373132	S-mmu-miR-142-3p-4373136	T-mmu-miR-142-3p-4373136
4	4	4	4
-0.157	-0.647	0.161	-0.577
-0.1245	-0.4418	0.249	-0.2452
0.042	0.2795	0.847	0.756
0.951	0.817	1.741	0.81
1.231	0.961	1.928	0.826
**0.2895**	**0.2183**	**0.9458**	**0.4403**
0.6371	0.6659	0.7796	0.679
0.3186	0.3329	0.3898	0.3395
-0.4601	-0.5651	0.02857	-0.3586
1.039	1.002	1.863	1.239
0	0	0	0
0.2895	0.2183	0.9458	0.4403
-0.2895	-0.2183	-0.9458	-0.4403
-0.4601 to 1.039	-0.5651 to 1.002	0.02857 to 1.863	-0.3586 to 1.239
t=0.9088 df=3	t=0.6555 df=3	t=2.426 df=3	t=1.297 df=3
0.4305	0.5589	0.0936	0.2854
1.158	0.873	3.783	1.761
0.4411		0.1829	

S-mmu-miR-140-4373374	T-mmu-miR-140-4373374	S-mmu-miR-139-5p-4395400	T-mmu-miR-139-5p-4395400	S-mmu-miR-139-3p-4395676	T-mmu-miR-139-3p-4395676
4	4	4	4	4	4
-0.266	-0.26	0.077	-0.232	-1.429	-2.736
-0.259	-0.1933	0.088	-0.1328	-1.04	-2.176
-0.1785	0.2465	0.155	0.212	0.3185	-0.333
0.6025	0.7673	0.1935	0.484	0.5763	-0.129
0.843	0.861	0.195	0.559	0.599	-0.115
**0.055**	**0.2735**	**0.1455**	**0.1878**	**-0.04825**	**-0.8793**
0.5292	0.4987	0.05667	0.3264	0.9427	1.249
0.2646	0.2493	0.02834	0.1632	0.4714	0.6246
-0.5676	-0.3132	0.07883	-0.1963	-1.157	-2.349
0.6776	0.8602	0.2122	0.5718	1.061	0.5903
0	0	0	0	0	0
0.055	0.2735	0.1455	0.1878	-0.04825	-0.8793
-0.055	-0.2735	-0.1455	-0.1878	0.04825	0.8793
-0.5676 to 0.6776	-0.3132 to 0.8602	0.07883 to 0.2122	-0.1963 to 0.5718	-1.157 to 1.061	-2.349 to 0.5903
t=0.2079 df=3	t=1.097 df=3	t=5.135 df=3	t=1.150 df=3	t=0.1024 df=3	t=1.408 df=3
0.8486	0.3528	0.0143	0.3333	0.9249	0.2539
0.22	1.094	0.582	0.751	-0.193	-3.517
0.2849		0.4036		0.1645	

S-mmu-miR-138-4395395	T-mmu-miR-138-4395395	S-mmu-miR-137-4373301	T-mmu-miR-137-4373301	S-mmu-miR-136-4395641	T-mmu-miR-136-4395641
4	4	4	4	4	4
-0.571	-0.404	-1.177	-0.542	-0.148	-0.126
-0.4598	-0.331	-0.9533	-0.3548	-0.1352	-0.06075
-0.0255	-0.0865	-0.016	0.2525	0.1655	0.152
0.3255	0.5173	0.715	0.7308	0.77	0.2515
0.409	0.71	0.87	0.875	0.884	0.279
**-0.05325**	**0.03325**	**-0.08475**	**0.2095**	**0.2668**	**0.1143**
0.4097	0.4758	0.8671	0.5818	0.4869	0.1716
0.2048	0.2379	0.4335	0.2909	0.2435	0.08578
-0.5352	-0.5265	-1.105	-0.475	-0.3061	-0.08758
0.4287	0.593	0.9354	0.894	0.8396	0.3161
0	0	0	0	0	0
-0.05325	0.03325	-0.08475	0.2095	0.2668	0.1143
0.05325	-0.03325	0.08475	-0.2095	-0.2668	-0.1143
-0.5352 to 0.4287	-0.5265 to 0.5930	-1.105 to 0.9354	-0.4750 to 0.8940	-0.3061 to 0.8396	-0.08758 to 0.3161
t=0.2600 df=3	t=0.1398 df=3	t=0.1955 df=3	t=0.7202 df=3	t=1.096 df=3	t=1.332 df=3
0.8117	0.8977	0.8575	0.5235	0.3533	0.275
-0.213	0.133	-0.339	0.838	1.067	0.457
0.3961		0.2967		0.2881	

S-mmu-miR-135b-4395372	T-mmu-miR-135b-4395372	S-mmu-miR-134-4373299	T-mmu-miR-134-4373299	S-mmu-miR-132-4373143	T-mmu-miR-132-4373143
4	4	4	4	4	4
-2.243	-1.524	-0.489	-0.352	-0.311	0.236
-2.016	-0.986	-0.3377	-0.269	-0.087	0.2442
0.107	0.9095	0.1555	-0.0155	0.9565	1.044
3.144	2.599	0.4493	0.3828	1.567	2.829
3.676	3.068	0.534	0.514	1.646	3.165
**0.4117**	**0.8408**	**0.089**	**0.03275**	**0.812**	**1.372**
2.711	1.89	0.4259	0.3579	0.8707	1.405
1.355	0.9452	0.2129	0.179	0.4354	0.7025
-2.778	-1.383	-0.412	-0.3883	-0.2124	-0.2807
3.601	3.065	0.59	0.4538	1.836	3.025
0	0	0	0	0	0
0.4117	0.8408	0.089	0.03275	0.812	1.372
-0.4117	-0.8408	-0.089	-0.03275	-0.812	-1.372
-2.778 to 3.601	-1.383 to 3.065	-0.4120 to 0.5900	-0.3883 to 0.4538	-0.2124 to 1.836	-0.2807 to 3.025
t=0.3038 df=3	t=0.8895 df=3	t=0.4180 df=3	t=0.1830 df=3	t=1.865 df=3	t=1.953 df=3
0.7812	0.4393	0.7041	0.8665	0.159	0.1458
1.647	3.363	0.356	0.131	3.248	5.489
0.4019		0.4232		0.2616	

S-mmu-miR-129-3p-4373297	T-mmu-miR-129-3p-4373297	S-mmu-miR-127-4373147	T-mmu-miR-127-4373147	S-mmu-miR-126-5p-4373269	T-mmu-miR-126-5p-4373269
4	4	4	4	4	4
-1.211	-2.433	-1.063	-1.155	-0.262	0.025
-1.156	-2.165	-0.934	-1.014	-0.2213	0.03725
-0.582	-1.094	0.0585	0.053	0.042	0.261
0.0405	-0.243	0.8065	0.9962	0.5385	1.194
0.112	-0.048	0.854	1.096	0.657	1.443
**-0.5658**	**-1.167**	**-0.023**	**0.01175**	**0.1198**	**0.4975**
0.6349	1.001	0.9305	1.06	0.4026	0.658
0.3174	0.5006	0.4652	0.5299	0.2013	0.329
-1.313	-2.345	-1.118	-1.235	-0.3539	-0.2767
0.1812	0.01064	1.072	1.259	0.5934	1.272
0	0	0	0	0	0
-0.5658	-1.167	-0.023	0.01175	0.1198	0.4975
0.5658	1.167	0.023	-0.01175	-0.1198	-0.4975
-1.313 to 0.1812	-2.345 to 0.01064	-1.118 to 1.072	-1.235 to 1.259	-0.3539 to 0.5934	-0.2767 to 1.272
t=1.782 df=3	t=2.332 df=3	t=0.04944 df=3	t=0.02217 df=3	t=0.5949 df=3	t=1.512 df=3
0.1727	0.102	0.9637	0.9837	0.5938	0.2277
-2.263	-4.669	-0.092	0.047	0.479	1.99
0.1747		0.4811		0.1826	

S-mmu-miR-126-3p-4395339	T-mmu-miR-126-3p-4395339	S-mmu-miR-125b-5p-4373148	T-mmu-miR-125b-5p-4373148	S-mmu-miR-125a-5p-4395309	T-mmu-miR-125a-5p-4395309
4	4	4	4	4	4
-0.526	-0.055	-0.223	-0.258	-0.53	-0.585
-0.4453	-0.0002501	-0.03575	-0.1295	-0.4478	-0.4463
-0.1365	0.2775	0.6235	0.3535	-0.1225	-0.0205
0.06125	0.4683	1.088	0.8695	0.1975	0.2845
0.105	0.494	1.21	1.009	0.278	0.383
**-0.1735**	**0.2485**	**0.5585**	**0.3645**	**-0.1243**	**-0.06075**
0.2667	0.2448	0.5952	0.5235	0.336	0.398
0.1334	0.1224	0.2976	0.2617	0.168	0.199
-0.4873	-0.03955	-0.1417	-0.2514	-0.5196	-0.529
0.1403	0.5365	1.259	0.9804	0.2711	0.4075
0	0	0	0	0	0
-0.1735	0.2485	0.5585	0.3645	-0.1243	-0.06075
0.1735	-0.2485	-0.5585	-0.3645	0.1243	0.06075
-0.4873 to 0.1403	-0.03955 to 0.5365	-0.1417 to 1.259	-0.2514 to 0.9804	-0.5196 to 0.2711	-0.5290 to 0.4075
t=1.301 df=3	t=2.030 df=3	t=1.877 df=3	t=1.393 df=3	t=0.7395 df=3	t=0.3053 df=3
0.2842	0.1353	0.1572	0.258	0.5132	0.7801
-0.694	0.994	2.234	1.458	-0.497	-0.243
0.0293		0.3209		0.4077	

S-mmu-miR-10b-4395329	T-mmu-miR-10b-4395329	S-mmu-miR-106b-4373155	T-mmu-miR-106b-4373155	S-mmu-miR-106a-4395589	T-mmu-miR-106a-4395589
4	4	4	4	4	4
-0.972	-1.275	-0.609	-1.579	0.139	-0.304
-0.6618	-1.045	-0.399	-1.3	0.1487	-0.2653
0.522	-0.3295	0.2335	-0.45	0.455	-0.029
1.551	-0.1115	0.254	0.6658	0.9158	0.1675
1.81	-0.047	0.26	1.034	0.977	0.193
**0.4705**	**-0.4953**	**0.0295**	**-0.3613**	**0.5065**	**-0.04225**
1.156	0.537	0.4259	1.072	0.4144	0.2258
0.578	0.2685	0.2129	0.5359	0.2072	0.1129
-0.8894	-1.127	-0.4715	-1.622	0.01895	-0.3079
1.83	0.1365	0.5305	0.8996	0.994	0.2234
0	0	0	0	0	0
0.4705	-0.4953	0.0295	-0.3613	0.5065	-0.04225
-0.4705	0.4953	-0.0295	0.3613	-0.5065	0.04225
-0.8894 to 1.830	-1.127 to 0.1365	-0.4715 to 0.5305	-1.622 to 0.8996	0.01895 to 0.9940	-0.3079 to 0.2234
t=0.8141 df=3	t=1.845 df=3	t=0.1385 df=3	t=0.6742 df=3	t=2.444 df=3	t=0.3742 df=3
0.4752	0.1623	0.8986	0.5485	0.0921	0.7332
1.882	-1.981	0.118	-1.445	2.026	-0.169
0.0902		0.2616		0.0295	

S-mmu-miR-103-4373158	T-mmu-miR-103-4373158	S-mmu-miR-101a-4395364	T-mmu-miR-101a-4395364	S-mmu-let-7i-4395332	T-mmu-let-7i-4395332
4	4	4	4	4	4
-0.216	0.356	-0.481	-0.509	-0.23	-0.049
-0.2043	0.392	-0.131	-0.3902	-0.1883	-0.0275
0.2695	0.5335	0.924	0.597	0.0195	0.32
0.945	1.163	1.354	1.516	0.4673	1.149
1.024	1.361	1.495	1.612	0.589	1.331
**0.3368**	**0.696**	**0.7155**	**0.5743**	**0.0995**	**0.4805**
0.6249	0.452	0.8419	1.008	0.3534	0.6365
0.3124	0.226	0.4209	0.504	0.1767	0.3183
-0.3984	0.1642	-0.275	-0.6116	-0.3162	-0.2683
1.072	1.228	1.706	1.76	0.5152	1.229
0	0	0	0	0	0
0.3368	0.696	0.7155	0.5743	0.0995	0.4805
-0.3368	-0.696	-0.7155	-0.5743	-0.0995	-0.4805
-0.3984 to 1.072	0.1642 to 1.228	-0.2750 to 1.706	-0.6116 to 1.760	-0.3162 to 0.5152	-0.2683 to 1.229
t=1.078 df=3	t=3.080 df=3	t=1.700 df=3	t=1.139 df=3	t=0.5632 df=3	t=1.510 df=3
0.3601	0.0541	0.1877	0.3372	0.6127	0.2282
1.347	2.784	2.862	2.297	0.398	1.922
0.1937		0.4184		0.1678	

S-mmu-let-7g-4395393	T-mmu-let-7g-4395393	S-mmu-let-7f-4373164	T-mmu-let-7f-4373164	S-mmu-let-7e-4395517	T-mmu-let-7e-4395517
4	4	4	4	4	4
-0.702	-0.175	-0.684	-1.474	-1.208	-0.808
-0.559	-0.0385	-0.481	-1.188	-1.14	-0.7315
0.118	0.82	0.254	0.194	-0.4595	-0.3955
0.5858	1.308	0.404	0.7805	0.7275	0.6388
0.659	1.321	0.412	0.802	0.965	0.948
**0.04825**	**0.6965**	**0.059**	**-0.071**	**-0.2905**	**-0.1628**
0.5968	0.7265	0.5114	1.067	0.9875	0.7705
0.2984	0.3632	0.2557	0.5335	0.4938	0.3853
-0.6539	-0.1582	-0.5426	-1.326	-1.452	-1.069
0.7504	1.551	0.6606	1.184	0.8713	0.7438
0	0	0	0	0	0
0.04825	0.6965	0.059	-0.071	-0.2905	-0.1628
-0.04825	-0.6965	-0.059	0.071	0.2905	0.1628
-0.6539 to 0.7504	-0.1582 to 1.551	-0.5426 to 0.6606	-1.326 to 1.184	-1.452 to 0.8713	-1.069 to 0.7438
t=0.1617 df=3	t=1.918 df=3	t=0.2308 df=3	t=0.1331 df=3	t=0.5884 df=3	t=0.4224 df=3
0.8818	0.151	0.8323	0.9026	0.5977	0.7012
0.193	2.786	0.236	-0.284	-1.162	-0.651
0.1086		0.4167		0.4226	

S-mmu-let-7d-4395394	T-mmu-let-7d-4395394	S-mmu-let-7c-4373167	S-mmu-let-7c-4373167	S-mmu-let-7b-4373168	T-mmu-let-7b-4373168
4	4	4	4	4	4
-0.403	-0.078	-0.063	-0.202	-0.229	-0.615
-0.2525	-0.04425	-0.02	-0.1863	-0.1628	-0.402
0.233	0.322	0.26	0.0405	0.0645	0.2475
0.6862	1.342	0.8003	0.394	0.7035	0.456
0.826	1.593	0.93	0.452	0.907	0.522
**0.2223**	**0.5398**	**0.3468**	**0.08275**	**0.2018**	**0.1005**
0.5027	0.7586	0.4354	0.3085	0.4907	0.4943
0.2513	0.3793	0.2177	0.1542	0.2453	0.2472
-0.3691	-0.3527	-0.1655	-0.2802	-0.3755	-0.4811
0.8136	1.432	0.859	0.4457	0.779	0.6821
0	0	0	0	0	0
0.2223	0.5398	0.3468	0.08275	0.2018	0.1005
-0.2223	-0.5398	-0.3468	-0.08275	-0.2018	-0.1005
-0.3691 to 0.8136	-0.3527 to 1.432	-0.1655 to 0.8590	-0.2802 to 0.4457	-0.3755 to 0.7790	-0.4811 to 0.6821
t=0.8843 df=3	t=1.423 df=3	t=1.593 df=3	t=0.5365 df=3	t=0.8224 df=3	t=0.4066 df=3
0.4417	0.2499	0.2095	0.6288	0.4711	0.7116
0.889	2.159	1.387	0.331	0.807	0.402
0.2557		0.1803		0.3905	

S-mmu-let-7a-4373169	T-mmu-let-7a-4373169	S-mmu-miR-133a-4395357	T-mmu-miR-133a-4395357	S-mmu-miR-130b-4373144	T-mmu-miR-130b-4373144
4	4	4	4	4	4
-0.096	-0.622	-0.872	-0.65	-1.435	-2.456
-0.0835	-0.345	-0.7878	-0.5383	-0.8835	-1.995
0.074	0.5275	-0.445	0.1255	1.182	0.688
1.095	1.378	-0.1338	2.158	2.525	2.01
1.395	1.648	-0.06	2.726	2.836	2.017
**0.3617**	**0.5203**	**-0.4555**	**0.5818**	**0.9413**	**0.2342**
0.7004	0.9274	0.3398	1.5	1.797	2.176
0.3502	0.4637	0.1699	0.7498	0.8986	1.088
-0.4622	-0.5708	-0.8552	-1.183	-1.173	-2.326
1.186	1.611	-0.05577	2.346	3.056	2.794
0	0	0	0	0	0
0.3617	0.5203	-0.4555	0.5818	0.9413	0.2342
-0.3617	-0.5203	0.4555	-0.5818	-0.9413	-0.2342
-0.4622 to 1.186	-0.5708 to 1.611	-0.8552 to -0.05577	-1.183 to 2.346	-1.173 to 3.056	-2.326 to 2.794
t=1.033 df=3	t=1.122 df=3	t=2.681 df=3	t=0.7758 df=3	t=1.047 df=3	t=0.2153 df=3
0.3776	0.3436	0.075	0.4944	0.3719	0.8433
1.447	2.081	-1.822	2.327	3.765	0.937
0.3971		0.113		0.3171	
