# Supplementary material for: An approach to identify microRNAs involved in neuropathic pain following a peripheral nerve injury
Source: Front Neurosci. 2014 Aug 29;8:266. doi: 10.3389/fnins.2014.00266 (PMC4148822; doi:10.3389/fnins.2014.00266)
Supplement: Supplementary file 2 [file DataSheet2.PDF]

Supplement 2

mmu-miR-133b-4395358

>mmu-miR-133b-3p MIMAT0000769 UUUGGUCCCCUUCAACCAGCUA
>hsa-miR-133b MIMAT0000770 UUUGGUCCCCUUCAACCAGCUA
>rno-miR-133b-3p MIMAT0003126 UUUGGUCCCCUUCAACCAGCUA

mmu-miR-145-4395389

>mmu-miR-145a-5p MIMAT0000157 GUCCAGUUUUCCCAGGAAUCCCU
>hsa-miR-145-5p MIMAT0000437 GUCCAGUUUUCCCAGGAAUCCCU
>rno-miR-145-5p MIMAT0000851 GUCCAGUUUUCCCAGGAAUCCCU

mmu-miR-193b-4395597

>mmu-miR-193b-3p MIMAT0004859 AACUGGCCCACAAAGUCCCGCU
>hsa-miR-193b-3p MIMAT0002819 AACUGGCCCUCAAAGUCCCGCU
>rno-miR-193b-3p MIMAT0035734 AACUGGCCC**A**CAAAGUCCC

mmu-miR-143-4395360

>mmu-miR-143-3p MIMAT0000247 UGAGAUGAAGCACUGUAGCUC
>hsa-miR-143-3p MIMAT0000435 UGAGAUGAAGCACUGUAGCUC
>rno-miR-143-3p MIMAT0000849 UGAGAUGAAGCACUGUAGCUCA

mmu-miR-335-5p-4373045

>mmu-miR-335-5p MIMAT0000766 UCAAGAGCAAUAACGAAAAAUGU
>hsa-miR-335-5p MIMAT0000765 UCAAGAGCAAUAACGAAAAAUGU
>rno-miR-335 MIMAT0000575 UCAAGAGCAAUAACGAAAAAUGU

mmu-miR-191-4395410

>mmu-miR-191-5p MIMAT0000221 CAACGGAAUCCCAAAAGCAGCUG
>hsa-miR-191-5p MIMAT0000440 CAACGGAAUCCCAAAAGCAGCUG
>rno-miR-191a-5p MIMAT0000866 CAACGGAAUCCCAAAAGCAGCUG

rno-miR-1-3p

>rno-miR-1-3p MIMAT0003125 UGGAAUGUAAAGAAGUGUGUAU
>mmu-miR-1a-3p MIMAT0000123 UGGAAUGUAAAGAAGU**A**UGUAU
>hsa-miR-1-3p MIMAT0000416 UGGAAUGUAAAGAAGU**A**UGUAU
